# Structural modifications toward improved lead-203/lead-212 peptide-based image-guided alpha-particle radiopharmaceutical therapies for neuroendocrine tumors

**DOI:** 10.1007/s00259-023-06494-9

**Published:** 2023-11-13

**Authors:** Dongyoul Lee, Mengshi Li, Dijie Liu, Nicholas J. Baumhover, Edwin A. Sagastume, Brenna M. Marks, Prerna Rastogi, F. Christopher Pigge, Yusuf Menda, Frances L. Johnson, Michael K. Schultz

**Affiliations:** 1 Department of Physics and Chemistry, Korea Military Academy, Seoul, Republic of Korea; 2 Perspective Therapeutics, Inc., Coralville, IA USA; 3 Department of Pathology, The University of Iowa Hospitals and Clinics, Iowa City, IA USA; 4 Department of Chemistry, The University of Iowa, ML B180 FRRBP, 500 Newton Road, Iowa City, IA 52240 USA; 5 Department of Radiology, The University of Iowa Hospitals and Clinics, Iowa City, IA USA; 6 Department of Radiation Oncology, The University of Iowa Hospitals and Clinics, Iowa City, IA USA

**Keywords:** Peptides, Structural optimization, Lead isotopes, Alpha-particle therapy, SSTR2, PRRT

## Abstract

**Purpose:**

The lead-203 (^203^Pb)/lead-212 (^212^Pb) elementally identical radionuclide pair has gained significant interest in the field of image-guided targeted alpha-particle therapy for cancer. Emerging evidence suggests that ^212^Pb-labeled peptide-based radiopharmaceuticals targeting somatostatin receptor subtype 2 (SSTR2) may provide improved effectiveness compared to beta-particle-based therapies for neuroendocrine tumors (NETs). This study aims to improve the performance of SSTR2-targeted radionuclide imaging and therapy through structural modifications to Tyr^3^-octreotide (TOC)-based radiopharmaceuticals.

**Methods:**

New SSTR2-targeted peptides were designed and synthesized with the goal of optimizing the incorporation of Pb isotopes through the use of a modified cyclization technique; the introduction of a Pb-specific chelator (PSC); and the insertion of polyethylene glycol (PEG) linkers. The binding affinity of the peptides and the cellular uptake of ^203^Pb-labeled peptides were evaluated using pancreatic AR42J (SSTR2+) tumor cells and the biodistribution and imaging of the ^203^Pb-labeled peptides were assessed in an AR42J tumor xenograft mouse model. A lead peptide was identified (i.e., PSC-PEG_2_-TOC), which was then further evaluated for efficacy in ^212^Pb therapy studies.

**Results:**

The lead radiopeptide drug conjugate (RPDC) — [^203^Pb]Pb-PSC-PEG_2_-TOC — significantly improved the tumor-targeting properties, including receptor binding and tumor accumulation and retention as compared to [^203^Pb]Pb-DOTA^0^-Tyr^3^-octreotide (DOTATOC). Additionally, the modified RPDC exhibited faster renal clearance than the DOTATOC counterpart. These advantageous characteristics of [^212^Pb]Pb-PSC-PEG_2_-TOC resulted in a dose-dependent therapeutic effect with minimal signs of toxicity in the AR42J xenograft model. Fractionated administrations of 3.7 MBq [^212^Pb]Pb-PSC-PEG_2_-TOC over three doses further improved anti-tumor effectiveness, resulting in 80% survival (70% complete response) over 120 days in the mouse model.

**Conclusion:**

Structural modifications to chelator and linker compositions improved tumor targeting and pharmacokinetics (PK) of ^203/212^Pb peptide-based radiopharmaceuticals for NET theranostics. These findings suggest that PSC-PEG_2_-TOC is a promising candidate for Pb-based targeted radionuclide therapy for NETs and other types of cancers that express SSTR2.

**Supplementary Information:**

The online version contains supplementary material available at 10.1007/s00259-023-06494-9.

## Introduction

NETs are a diverse group of neoplasms that can originate and occur in many different organs and tissues of the body [1]. Although disease progression can be variable, these tumors often progress slowly and may be asymptomatic or accompanied by non-specific symptoms, which can make them difficult to diagnose [2, 3]. As a result, NET patients often have inoperable tumors or metastases at the time of diagnosis [1, 4], which necessitates identification of an effective systemic therapy.

One such class of therapy is SSTR2-targeted peptide receptor radionuclide therapy (PRRT). Therapeutic outcomes with the current standard-of-care PRRT, lutetium-177-DOTATATE {[^177^Lu]Lu-DOTATATE; Lutathera^®^} targeting SSTR2, have been shown to be effective for well-differentiated gastroenteropancreatic (GEP) NETs [5, 6]. However, despite the promise of the beta-particle-based PRRT (b-PRRT), objective responses are seen in only 18–30% of patients with midgut NETs with 1% complete response rate, emphasizing the critical need for improved therapy options [5, 6]. Preclinical [7–12] and clinical [13–16] evidence suggests that alpha-particle-based PRRT (a-PRRT) may be more effective than b-PRRT for NETs. ^212^Pb is an attractive in vivo a-particle generator for targeted-radiopharmaceutical therapy. The half-life (*t*_1/2_=10.6 h) of ^212^Pb matches well with the biological half-lives of small peptides [17], with potent a-particle emissions in the decay series [18–20]. In addition, the availability of elementally identical ^203^Pb provides an imaging surrogate for ^212^Pb with decay properties (*t*_1/2_ = 52 h; 279 keV gamma ray; 81% intensity) that allow for multiple time point single-photon emission computed tomography (SPECT and SPECT/CT). This property enables the potential for precise assessment of radiopharmaceutical pharmacokinetic (PK) properties not only in the preclinical development phase, but also in the clinical setting for determination of image-derived individualized normal organ and tumor dosimetry-based treatment plans.

In this context, molecular modifications to peptide structures can dramatically change the binding affinities to targeted receptors, PK properties, and the biodistribution of peptides for radiopharmaceutical applications — with the goal of ultimately improving the therapeutic window (and outcomes) of the radiopharmaceutical [21]. Potential structural modifications that can affect performance characteristics include changes in amino acid sequence, cyclization of the peptide sequence with biologically stable chemical features, insertion of a chemical linker between the chelator and the peptide backbone (optimized for length and chemical composition), and development of radionuclide-specific chelators that optimize radiolabeling efficiency and radiometal chelator coupling. DOTATOC and DOTATATE, the most widely used peptides targeting SSTR2, are cyclized with a disulfide bond to maintain their structural integrity. However, the disulfide bond and (more significantly) sulfhydryl groups after cleavage are known to increase non-specific accumulation of the peptides in the kidneys [22, 23]. Thus, it can be hypothesized that replacing the disulfide bond with other synthetic cyclization approaches, such as the so-called click chemistry [24], may reduce kidney uptake and improve in vivo stability. The choice of chelator is also an important structural consideration for RPDC design. For example, changes to the chelator structure and the selection of the radioisotope often lead to alterations in tumor uptake, normal organ clearance, and other PK properties [25, 26]. Additionally, the chemistry of the linker that connects the chelator to the peptide backbone represents another key bioconjugate tool that can be used to alter the PK of the RPDC [27–31]. Within this context, the performance of the ^203^Pb/^212^Pb theranostic pair in combination with different chelators, cyclization techniques, and chemical linkers has not been systematically tested for SSTR2-targeted therapy in NETs.

In this study, we aimed to develop a new form of peptide bioconjugate, specifically designed for the inclusion of Pb isotopes with the goal of improving SSTR2 targeting in NET models. We used various synthetic design strategies, such as click chemistry-based cyclization, a new chelator composition (1,4,7,10-tetraazacyclododecane-7-acetamide-1,4,10-triacetic acid, herein referred to as the Pb-specific chelator or PSC), and PEG linker insertions, to optimize tumor accumulation and retention while improving renal clearance.

## Materials and methods

### Peptide synthesis and radiochemistry

**Fig. 1:**
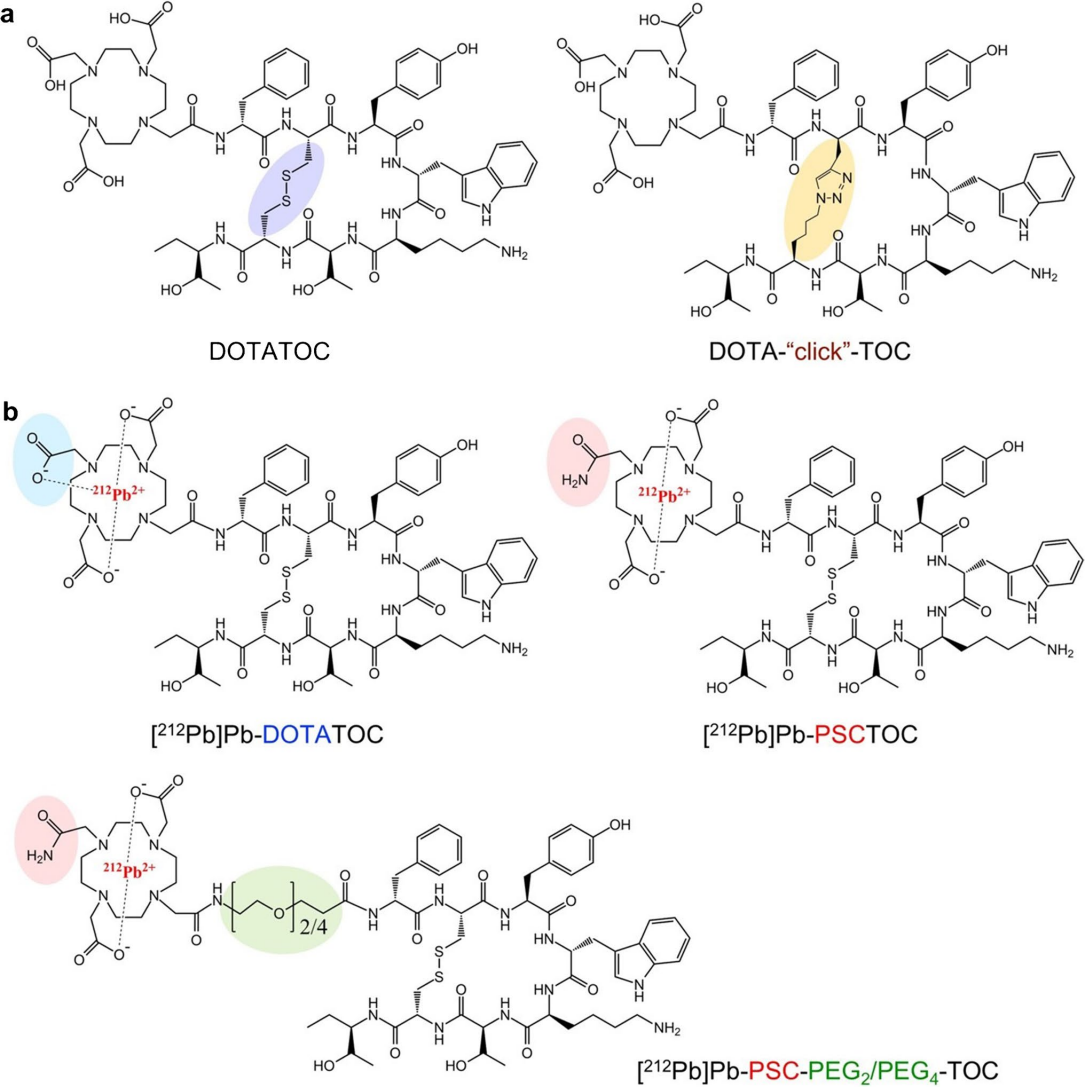
Structural modifications based on TOC for potential improvement of SSTR2-targeted therapeutic performance. The strategies included **a** a click cyclization approach to substitute the disulfide bond with a triazole link and **b** installation of a Pb-specific structural optimization of TOC with the incorporation of a new chelator composition, Pb-specific chelator (PSC), and various sizes of polyethylene glycol (PEG) linkers

New peptides were designed and synthesized using standard fluorenylmethyloxycarbonyl (Fmoc)-based solid phase peptide synthesis. The peptides were developed under two basic strategies: (a) a click cyclization approach to substitute the disulfide bond of TOC with a triazole link, which potentially improves PK and in vivo stability; and (b) Pb-specific structural optimization based on TOC by incorporating the new chelator composition and various sizes of PEG linkers (Fig. 1 and Table 1). To wit, peptide conjugates DOTATOC, click-cyclized DOTATOC (DOTA-click-TOC), PSCTOC, PSC-PEG_2_-TOC, and PSC-PEG_4_-TOC were synthesized and evaluated. The radiolabeling and purification process for the preparation of ^203^Pb/^212^Pb-labeled peptides followed the methods outlined in a previously published study [32]. The details of peptide synthesis, ^212^Pb dose calibration, ^203^Pb/^212^Pb radiolabeling, and dose preparation are described in the Supplementary Material. The stability of the most promising RPDC, [^203^Pb]Pb-PSC-PEG_2_-TOC, was evaluated in water and normal human serum up to 24 h of incubation at 37 °C using the radio-HPLC system. Furthermore, PSC-PEG_2_-TOC was assessed for a radiolabeling yield with clinically relevant ^203^Pb activity (1.8–3.6 GBq) to assess changes in performance with increases in molar activity (MBq/nmol; Supplementary Material).

**Table 1:** Overview of the characteristics of the TOC-based peptide variants

A.Peptide	Sequence	Mass (obs./calc.)	HPLC retention, min (unlabeled/Pb-labeled)^a^	IC_50_ (nM)
TOC	D-Phe-cyclo[Cys-Tyr-D-Trp-Lys-Thr-Cys]-Thr-OH	1034.4/1034.4	ND/NA	3.1 ± 1.1
DOTATOC	DOTA-D-Phe-cyclo[Cys-Tyr-D-Trp-Lys-Thr-Cys]-Thr-OH	1420.8/1420.6	15.3/18.0	11.3 ± 1.3
DOTA-click-TOC	DOTA-D-Phe-cyclo[Pra-Tyr-D-Trp-Lys-Thr-Lys(N_3_)]-Thr-OH	1465.8/1465.7	16.7/ND	ND
PSCTOC	PSC-D-Phe-cyclo[Cys-Tyr-D-Trp-Lys-Thr-Cys]-Thr-OH	1419.3/1419.6	14.8/16.9	6.2 ± 1.1
PSC-PEG_2_-TOC	PSC-PEG_2_-D-Phe-cyclo[Cys-Tyr-D-Trp-Lys-Thr-Cys]-Thr-OH	1578.8/1578.7	17.5/19.0	5.3 ± 1.2
PSC-PEG_4_-TOC	PSC-PEG_4_-D-Phe-cyclo[Cys-Tyr-D-Trp-Lys-Thr-Cys]-Thr-OH	1666.8/1666.8	19.8/21.3	9.4 ± 1.3

^a^The retention time for each peptide was evaluated in an HPLC system (Agilent 1200 Series) with a linear 16–26% acetonitrile (ACN) gradient in 20 mM HCl over 20 min (which initiated with 5-min equilibrium in 4% ACN followed by the increase to 16% in 1 min) at 37 °C on a Vydac 218TP C18 column (4.6×150 mm, 5 μm) with 1mL/min flow rate. *obs.*, observed; *calc.*, calculated; *ND*, not determined; *NA*, not applicable

### In vitro competitive binding assay

[^125^I]I-TOC was synthesized in-house by radiolabeling 37 MBq Na^125^I with 20 nmol TOC peptide via the Chloramine-T method as described by de Blois and colleagues in 2012 [33] and purified on reverse phase Strata-X C-18 column (Phenomenex, Torrance, CA, USA). SSTR2-positive AR42J rat pancreatic acinar cells were purchased from the American Type Culture Collection (ATCC; CRL-1492) and were cultured in RPMI 1640 medium, supplemented with 10% fetal bovine serum (FBS), 2 mM glutamine, and antibiotics (0.1 mg/mL streptomycin and 100 IU penicillin) at 37 °C under a humidified condition (5% CO_2_). The cells were plated into poly-D-lysine-coated 24-well plates at a density of 1.0 × 10^5^ cells per well. At day 3, the cells were treated with 0.5 kBq of [^125^I]I-TOC and increasing concentrations (10^−11^ to 10^−6^ M) of the synthesized peptides (i.e., TOC, click-TOC, DOTATOC, PSCTOC, PSC-PEG_2_-TOC, or PSC-PEG_4_-TOC); treatment was conducted in binding medium (RPMI 1640 supplemented with 0.2% BSA and 0.3 mM 1,10-phenanthroline) for 2 h at 37 °C. Cells were then washed twice with ice-cold PBS and lysed with 0.5 N NaOH. The radioactivities of the harvested samples were measured using an automated gamma counter (PerkinElmer Cobra II; PerkinElmer, Freemont, CA). IC_50_ was determined using GraphPad Prism V8.0.

### Cellular uptake of ^203^Pb-labeled peptides

PSCTOC and PSC-PEG_2_-TOC were labeled with ^203^Pb and purified via HPLC for high molar activities as descibed in the Supplementary Material and in Li and collaborators [32]. The HPLC-purified ^203^Pb-labeled peptides (3.4 kBq) were incubated with AR42J cells in 24-well plates (pre-coated with poly-D-lysine), which were plated at a density of 2.0 × 10^5^ cells 2 days prior, in the binding medium at 37 °C for up to 120 min. The cells were then washed twice and lysed, and the radioactivities of the samples were determined (*n*=3).

### In vivo biodistribution of ^203^Pb-labeled peptides

All animal experiments were performed according to approved protocols that were compliant to all rules and regulations of federal regulatory bodies and the Institutional Animal Care and Use Committee (IACUC) at the University of Iowa.

AR42J cells (5.0 × 10^6^ cells/animal) were implanted subcutaneously on the left shoulder of female athymic nu/nu mice. 37 kBq of ^203^Pb-labeled DOTATOC, PSCTOC, or PSC-PEG_2_-TOC (molar activity: 22 MBq/nmol) was administered to the the tumor-bearing mice via tail vein. Extra groups of mice were co-administered 37 kBq of ^203^Pb-PSC-PEG_2_-TOC with DL-lysine (400 mg/kg) to determine the effect of amino acid on renal uptake of the radiopharmaceutical. Mice (*n*=3 per time point) were euthanized at 1, 3, and 24 h post-administration, and tumors and organs/tissues of interest were excised and weighed. The radioactivities of the samples were measured using the gamma counter.

### SPECT/CT imaging of [^203^Pb]Pb-DOTATOC vs. [^203^Pb]Pb-PSC-PEG_2_-TOC

Mice bearing AR42J xenografts (~ 400 mm^3^) were administered 11.1 MBq of [^203^Pb]Pb-DOTATOC or [^203^Pb]Pb-PSC-PEG_2_-TOC (molar activity: 62 MBq/nmol) with or without co-injection of excess (30 nmol) unlabeled PSC-PEG_2_-TOC. Serial SPECT/CT imaging was conducted at 3 h and 24 h post-administration. The imaging study was performed at the University of Iowa Small Animal Imaging Core using an INVEON trimodality SPECT/positron emission tomography (PET)/CT scanner (Siemens Preclinical, Knoxville, TN) equipped with medium-energy (0.3 mm) pinhole collimators. Data were reconstructed using three-dimensional ordered-subsets expectation maximization (OSEM-3D) algorithm with eight iterations and six subsets. Post-reconstruction images were smoothed with a 3D Gaussian kernel. Post-imaging biodistribution of the radiotracer in mice was obtained at 30 h post-administration.

### Tumor and kidney dosimetry

The Particle and Heavy Ion Transport code System (PHITS) [34] was utilized for dose calculations in the kidneys and tumor. The ^203^Pb biodistribution data were used as a surrogate to interpret the PK of the ^212^Pb-labeled peptides. The PK of the radiopharmaceutical was assumed to follow a mono-exponential curve. The details of dosimetry analyses are described in the Supplementary Material.

### [^212^Pb]Pb-PSC-PEG_2_-TOC alpha-particle radiotherapy

When the average tumor size of AR42J tumor-bearing mice reached a volume of about 150 mm^3^ (approximately 10 days after inoculation), 0.37 MBq and 1.85 MBq of [^212^Pb]Pb-PSC-PEG_2_-TOC (9 MBq/nmol) in 100 μL saline were administered to the mice. The administered activities were determined in reference to dosimetry analysis, in which a dose of 1.7 MBq was estimated to result in 5.4 Gy renal dose (Table 2). 1.85 MBq was anticipated to deposit a dose of about 6.0 Gy in the kidneys, which is expected to be safe for mice. After the initial study that demonstrated high therapeutic efficacy without significant toxicity, the dose was escalated in the subsequent study to 3.7 MBq of [^212^Pb]Pb-PSC-PEG_2_-TOC to investigate if the higher administered activity could be tolerated in mice and if further improvement on tumor control could be achieved. Mouse bodyweights and tumor sizes were measured at least twice per week for 90 days. The endpoints of the study included tumor size over 1500 mm^3^, tumor ulceration, bodyweight loss over 20%, and observed general toxicity.

**Table 2:** Estimated tumor and kidney doses and the maximum administration activity based on different renal dose limits in AR42J tumor-bearing nude mice

Radiopeptide	Estimated dose (Gy/MBq)			Maximum administration activity (MBq)^a^			Tumor dose at 5.4 Gy renal limit (Gy)
	Tumor	Kidney	T/K	5.4 Gy limit	11 Gy limit	20 Gy limit	
[^212^Pb]Pb-DOTATOC	2.43	7.03	0.35	0.77	1.57	2.85	1.87
[^212^Pb]Pb-PSCTOC	9.19	5.41	1.70	1.00	2.04	3.70	9.18
[^212^Pb]Pb-PSC-PEG_2_-TOC	12.70	6.22	2.04	0.87	1.77	3.22	11.03
[^212^Pb]Pb-PSC-PEG_2_-TOC (+ Lysine)	8.65	3.24	2.67	1.67	3.39	6.17	14.40

^a^Maximum administration activity was determined based on the renal dose limit of 5.4 Gy, 11 Gy, and 20 Gy. 5.4 Gy (27 Gy/5) is based on the safe levels of renal limit values from beta particles (27 Gy) and an RBE of 5 for alpha particles. 11 Gy and 20 Gy were based on a survival study in mice using [^213^Bi]Bi-DOTATATE and they represent LD_5_ and LD_50_ for 90 days respectively

In another study, possible treatment regimens of [^212^Pb]Pb-PSC-PEG_2_-TOC were determined by comparing single and fractionated administrations of [^212^Pb]Pb-PSC-PEG_2_-TOC in mice bearing AR42J tumors. When the average size of AR42J tumors in mice reached 130 mm^3^, the mice were administered a single dose (3.7 MBq on day 0) or fractionated doses (1.2 MBq × 3 cycles on day 0, day 9, and day 28) of [^212^Pb]Pb-PSC-PEG_2_-TOC. The mice were monitored up to 120 days. DL-lysine (400 mg/kg) was co-administered in 10 mice per each group for both studies.

Mouse individual tumor growth curves and bodyweight changes were recorded, and median survival in the format of Kaplan-Meier survival curves was determined using the GraphPad Prism (V8.0). The statistical significance of survival data was determined using the log-rank (Mantel-Cox) test.

## Results

### Structural modifications dramatically improve binding affinity to SSTR2 and cellular uptake

**Fig. 2:**
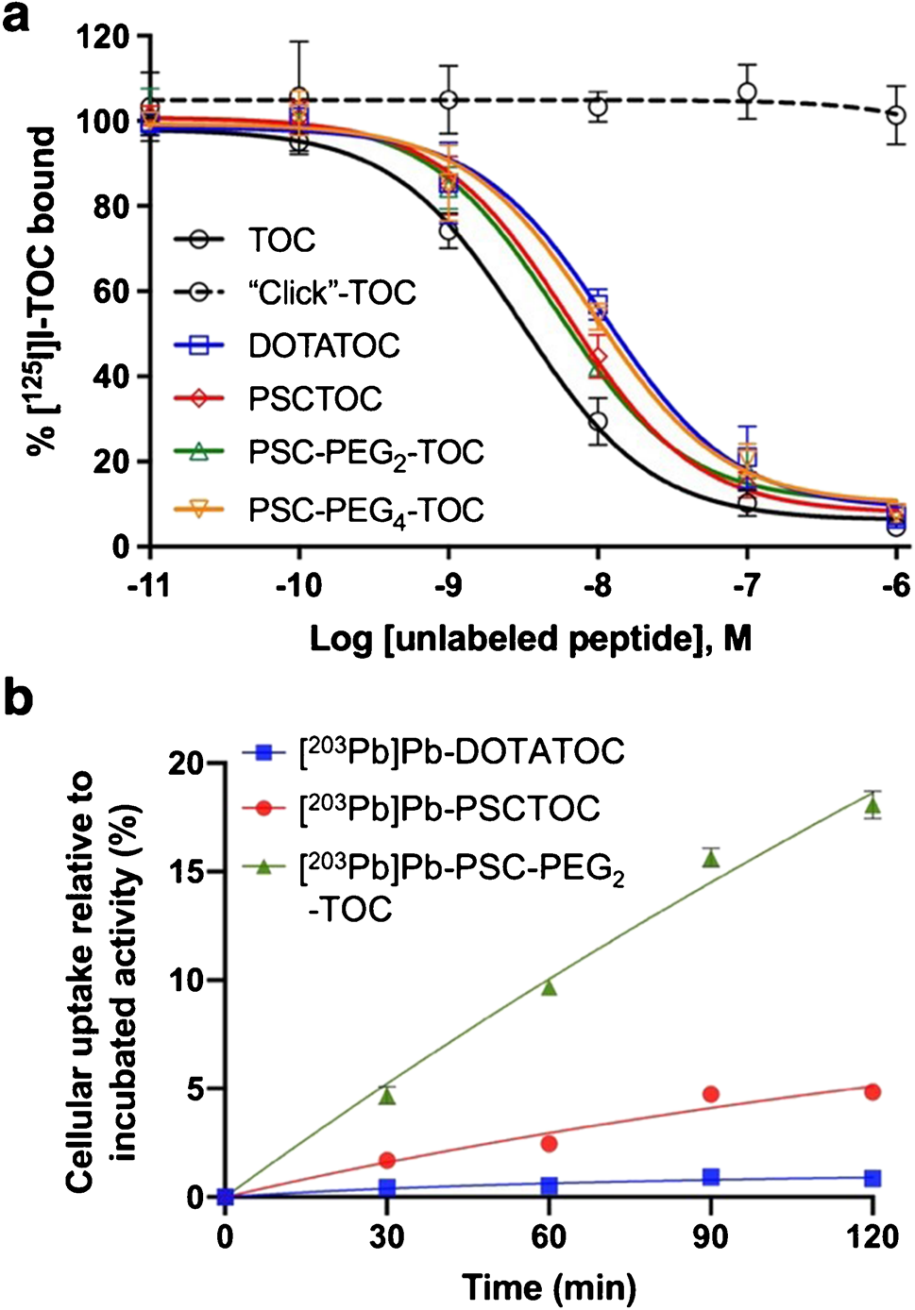
In vitro binding performance metrics of the newly synthesized peptides using AR42J SSTR2-positive cells. **a** Competitive inhibition plots of [^125^I]I-TOC binding to SSTR2 in AR42J cells with increasing concentrations of TOC and its structural variants. IC_50_ values; TOC: 3.1 ± 1.1 nM, DOTATOC: 11.3 ± 1.3 nM, PSCTOC: 6.2 ± 1.1 nM, PSC-PEG_2_-TOC: 5.3 ± 1.2 nM, PSC-PEG_4_-TOC: 9.4 ± 1.3 nM (at least *n*=6 from three biological replicates for DOTATOC and PSCTOC; *n*=4–6 from two biological replicates for TOC, “click” TOC, PSC-PEG_2_-TOC, and PSC-PEG_4_-TOC). **b** Cellular uptake of ^203^Pb-labeled DOTATOC, PSCTOC, and PSC-PEG_2_-TOC in AR42J cells. The binding medium, which contained 3.4 kBq of HPLC-purified 203Pb-labeled peptides, was incubated with the cells at 37 °C for up to 120 min

In vitro data suggested that click-cyclization of TOC significantly compromised the binding affinity to SSTR2 (Fig. 2a). Click-TOC did not competitively reduce the binding of [^125^I]I-TOC to SSTR2 in the 10^−11^ to 10^−6^ M concentration range.

Compared to DOTA chelator, the novel chelating agent composition (i.e., PSC) improved in vitro binding affinity to SSTR2 in AR42J cells when conjugated to TOC (Fig. 2a and Table 1). DOTATOC exhibited reduced binding affinity compared to TOC, but PSCTOC partially restored the affinity (IC_50_ values, TOC: 3.1 ± 1.1 nM vs. DOTATOC: 11.3 ± 1.3 nM vs. PSCTOC: 6.2 ± 1.1 nM). PSC-PEG_2_-TOC produced an improvement in affinity (5.3 ± 1.2 nM) vs. DOTATOC of more than a factor of 2, whereas PSC-PEG_4_-TOC provided an improved affinity than DOTATOC but weaker affinity (9.4 ± 1.3 nM) than PSCTOC.

[^203^Pb]Pb-PSCTOC exhibited a 6-fold improvement in cellular uptake compared to [^203^Pb]Pb-DOTATOC at 2 h post-incubation (Fig. 2b), demonstrating the potential improvements to SSTR2 binding that can be achieved by molecular modifications to the chelator for TOC bioconjugates. Moreover, [^203^Pb]Pb-PSC-PEG_2_-TOC showed remarkably (and significantly) higher cellular uptake than [^203^Pb]Pb-PSCTOC and [^203^Pb]Pb-DOTATOC by factors of 4 and 21 respectively, at the same incubation time (Fig. 2b).

Despite the comparable binding affinities (5.3 ± 1.2 nM vs. 6.2 ± 1.1 nM; Fig. 2a and Table 1) of PSC-PEG_2_-TOC (unlabeled) and PSCTOC (unlabeled), a large differential uptake was observed between these two peptides when labeled with ^203^Pb (5% for [^203^Pb]Pb-PSCTOC vs. 18% for [^203^Pb]Pb-PSC-PEG_2_-TOC at 2 h post-incubation). Taken together, the data show that the ^203^Pb-chelator complex hindered the receptor binding of TOC to a certain degree and that the addition of PEG_2_ linker minimized the effect of the Pb-chelator complex on receptor binding — and correspondingly restored the high affinity binding of TOC to SSTR2.

### [^203^Pb]Pb-PSC-PEG_2_-TOC demonstrated improved in vivo biodistribution profile

**Fig. 3:**
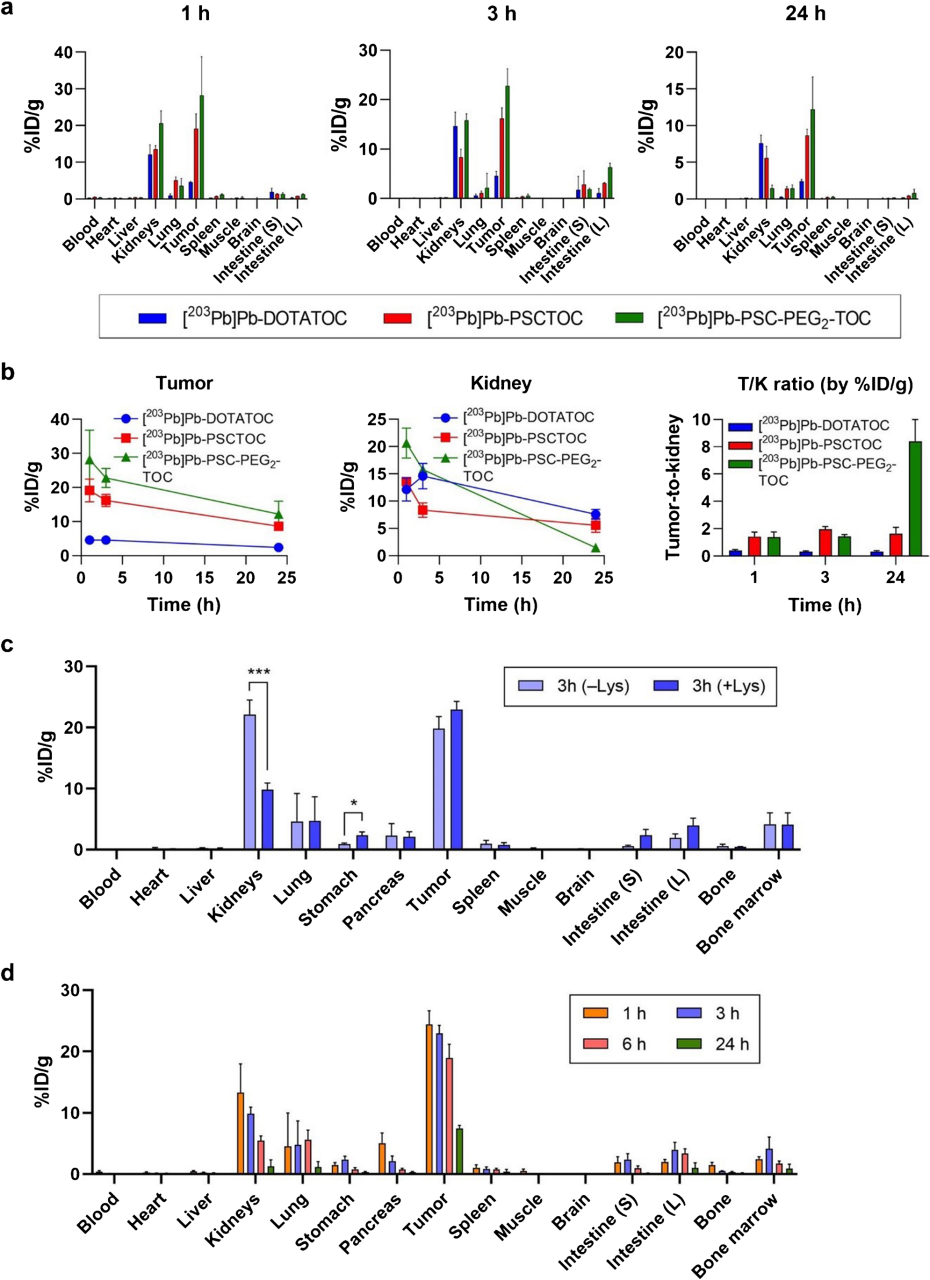
**a**–**b** Biodistribution of 203Pb-labeled DOTATOC, PSCTOC, and PSC-PEG_2_-TOC in AR42J tumor-bearing nude mice and %ID/g of tumor and kidneys as well as tumor-to-kidney ratios over time; **c** biodistribution of [203Pb]Pb-PSC-PEG_2_-TOC at 3 h post-administration in mice with and without co-administration of DL-lysine (400 mg/kg); and **d** comprehensive biodistribution of [203Pb]Pb-PSC-PEG_2_-TOC with DL-lysine co-administration at 1, 3, 6, and 24 h post-administration. The data are presented as mean values ± SD (*n*=3; *P<0.05, ***P<0.005). Intestine (S), small intestine; intestine (L), large intestine

The biodistribution of the TOC-based peptides dominated mainly by tumor accumulation/retention and renal excretion, with relatively low uptake in other tissues of interest (Fig. 3a). The in vivo tumor uptake exhibited a similar trend as was observed in the in vitro cellular uptake studies, where tumor uptake was highest for [^203^Pb]Pb-PSC-PEG_2_-TOC, followed by [^203^Pb]Pb-PSCTOC and [^203^Pb]Pb-DOTATOC (Fig. 3; Tables S1–S3). Notably, the [^203^Pb]Pb-PSC-PEG_2_-TOC cleared through the kidneys more rapidly than other peptides. The %ID/g values in the kidneys were significantly higher for [^203^Pb]Pb-PSC-PEG_2_-TOC than for [^203^Pb]Pb-DOTATOC at early time points (e.g., 20.6 %ID/g vs. 12.2 %ID/g, respectively at 1 h post-administration; Table S1), indicating an initial faster blood clearance via renal pathway rather than hepatobiliary system. The values became significantly lower for [^203^Pb]Pb-PSC-PEG_2_-TOC than for [^203^Pb]Pb-DOTATOC at 24 h (1.5 %ID/g vs. 7.6 %ID/g respectively; Table S3). The benefits of higher tumor uptake and faster renal clearance of the new peptide improved the tumor-to-kidney ratio substantially (Fig. 3b). In addition, co-administration of DL-lysine with the radiotracer significantly reduced the renal accumulation of [^203^Pb]Pb-PSC-PEG_2_-TOC by 56% without compromising the tumor uptake of the radiotracer in mice (Fig. 3c). A comprehensive biodistribution of [^203^Pb]Pb-PSC-PEG_2_-TOC with co-administration of DL-lysine was acquired at four different time points (1, 3, 6, and 24 h) in the mouse model (Fig. 3d). These data were subsequently used to interpret the PK of [^212^Pb]Pb-PSC-PEG_2_-TOC in the presence of DL-lysine for dosimetry analysis.

**Fig. 4:**
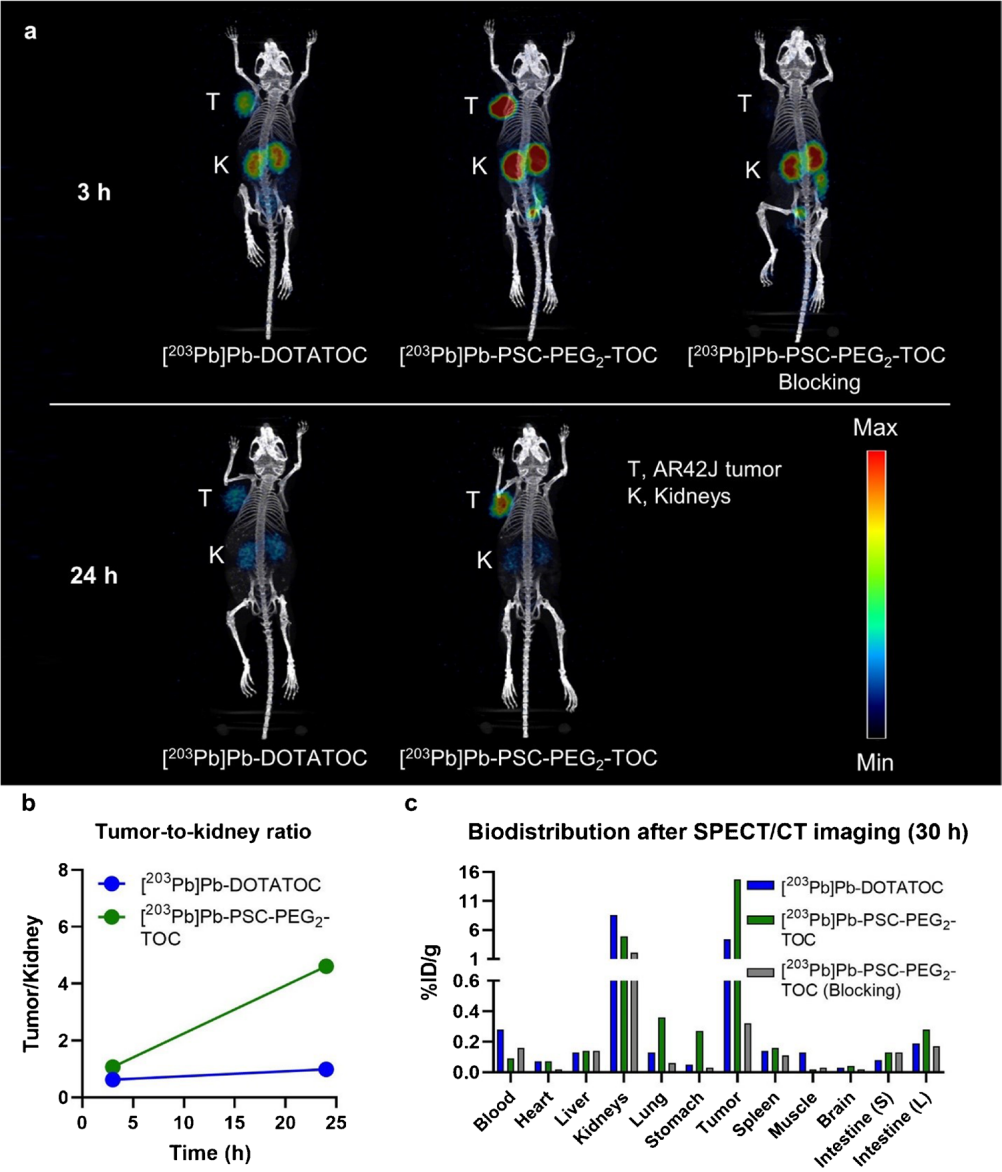
SPECT/CT imaging of [^203^Pb]Pb-DOTATOC and [^203^Pb]Pb-PEG_2_-TOC in AR42J tumor-bearing nude mice. **a** The AR42J-bearing mice were imaged at 3 h and 24 h after the administration of 11.1 MBq [^203^Pb]Pb-DOTATOC and [^203^Pb]Pb-PSC-PEG_2_-TOC. 30 nmol of cold peptide was co-administered for the blocking imaging to confirm the tumor specificity; **b** tumor-to-kidney uptake ratio over time, analyzed from the obtained images using the Inveon research workplace software; and **c** the mice were euthanized after the imaging study, and the biodistribution of [^203^Pb]Pb-PSC-PEG_2_-TOC was obtained at 30 h post-administration. Intestine (S), small intestine; intestine (L), large intestine

The potential of PSC-PEG_2_-TOC as a superior imaging agent linked to Pb isotopes was also observed by SPECT/CT imaging (Fig. 4). The SPECT/CT images obtained at 3 h and 24 h post-administration showed that [^203^Pb]Pb-PSC-PEG_2_-TOC had higher tumor uptake and faster renal clearance compared to [^203^Pb]Pb-DOTATOC, which increases the therapeutic index significantly (Fig. 4a, b). The tumor uptake of [^203^Pb]Pb-PSC-PEG_2_-TOC was almost completely blocked by co-administration of excessive unlabeled peptide, indicating that the high tumor uptake of [^203^Pb]Pb-PSC-PEG_2_-TOC is mediated by specific binding to SSTR2 (Fig. 4a, c).

### Dosimetry analysis of tumor and kidney dose deposition

**Fig. 5:**
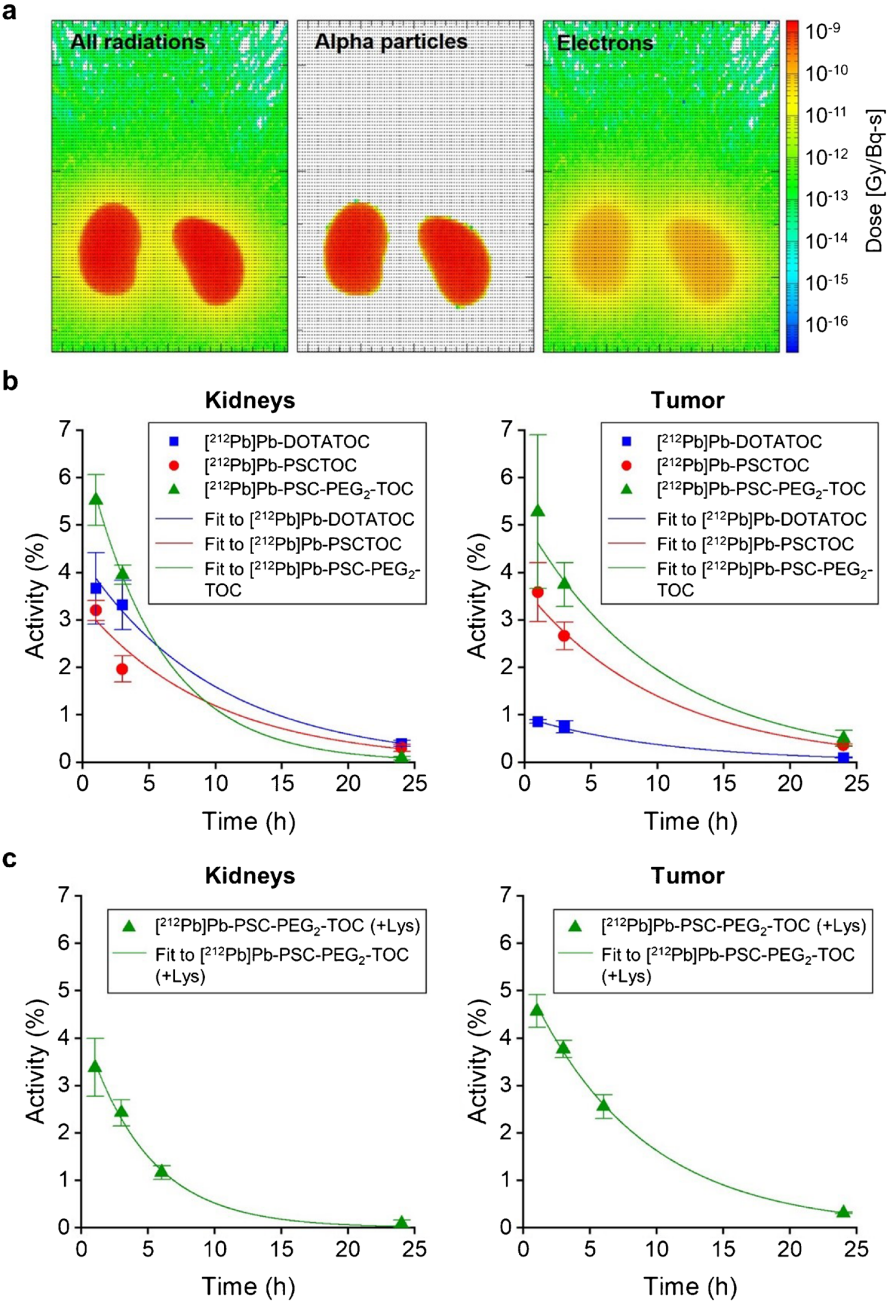
Dose deposition analysis in tumors and kidneys resulting from in vivo administration of ^212^Pb-labeled DOTATOC, PSCTOC, and PSC-PEG_2_-TOC. **a** Dose deposits from ^212^Pb decay in the kidneys of DigiMouse model; **b** time-activity curves of ^212^Pb-labeled peptides in tumor and kidneys of female nude mice, extrapolated from 203Pb biodistribution data; and **c** time-activity curves of [^212^Pb]Pb-PSC-PEG_2_-TOC with the co-administration of DL-lysine (400 mg/kg) in tumor and kidneys of female nude mice. Analysis was performed using single-phase exponential decay fitting with weighted function

Dosimetry analyses were conducted to estimate the dose absorbed in the tumor and the kidneys (Fig. 5). Based on the observed biodistribution, as expected the PHITS simulation platform (utilizing the DigiMouse voxel phantom model) identified kidneys as a dose limiting organ. Not surprisingly, radiation dose was dominated by alpha-particles (>93% of final dose) relative to beta particle emissions in the ^212^Pb decay series (Fig. 5a). Based on the ^203^Pb biodistribution data, the activity-curves were determined for ^212^Pb-labeled peptides with and without DL-lysine co-administration (Fig. 5b, c).

According to our analysis, the candidate — [^212^Pb]Pb-PSC-PEG_2_-TOC — exhibited the best ratio of radiation dose between the tumor and the kidneys. It scored 0.35, 1.70, and 2.04 for [^212^Pb]Pb-DOTATOC, [^212^Pb]Pb-PSCTOC, and [^212^Pb]Pb-PSC-PEG_2_-TOC, respectively, when we administered the same amount of radioactivity (Table 2). The data support that [^212^Pb]Pb-PSC-PEG_2_-TOC could deposit 20% more dose than [^212^Pb]Pb-PSCTOC to tumor with the same renal dose deposition. Compared to the renal doses resulting from [^212^Pb]Pb-PSC-PEG_2_-TOC in the absence of DL-lysine, the renal doses from [^212^Pb]Pb-PSC-PEG_2_-TOC in the presence of DL-lysine were estimated to be 48% lower. These data represent a 24% increase in tumor-specific dose according to the maximum administered radioactivity (Table 2).

Because the renal-dose limit for alpha-particles has not been established, the maximum administered radioactivity was estimated based on potential renal-dose limits from alpha-particles — 5.4 Gy, 11 Gy, and 20 Gy. 5.4 Gy was determined by 27 Gy, the renal-dose limit for beta particles [35, 36], divided by RBE = 5; 11 Gy and 20 Gy were based on the renal doses, which resulted in LD_5_ (lethal for 5% population by 90 days) and LD_50_ (lethal for 50% population by 90 days) respectively in nude mice, according to a survival study with [^213^Bi]Bi-DOTATATE [12]. The rightmost column in the Table 2 describes the estimated tumor dose deposited when the mice were maximally administered activities that can limit the dose to 5.4 Gy of alpha dose in the kidneys. In this analysis, PSC-PEG_2_-TOC can be administered about 13% more activity than DOTATOC (0.87 MBq/0.77 MBq = 1.13; based on 5.4 Gy renal-dose limit), and 5.9-fold higher absorbed dose can be deposited into the tumor with the maximum activity (11.03 Gy vs. 1.87 Gy).

### [^212^Pb]Pb-PSC-PEG_2_-TOC radiotherapy demonstrated robust anti-tumor effectiveness

**Fig. 6:**
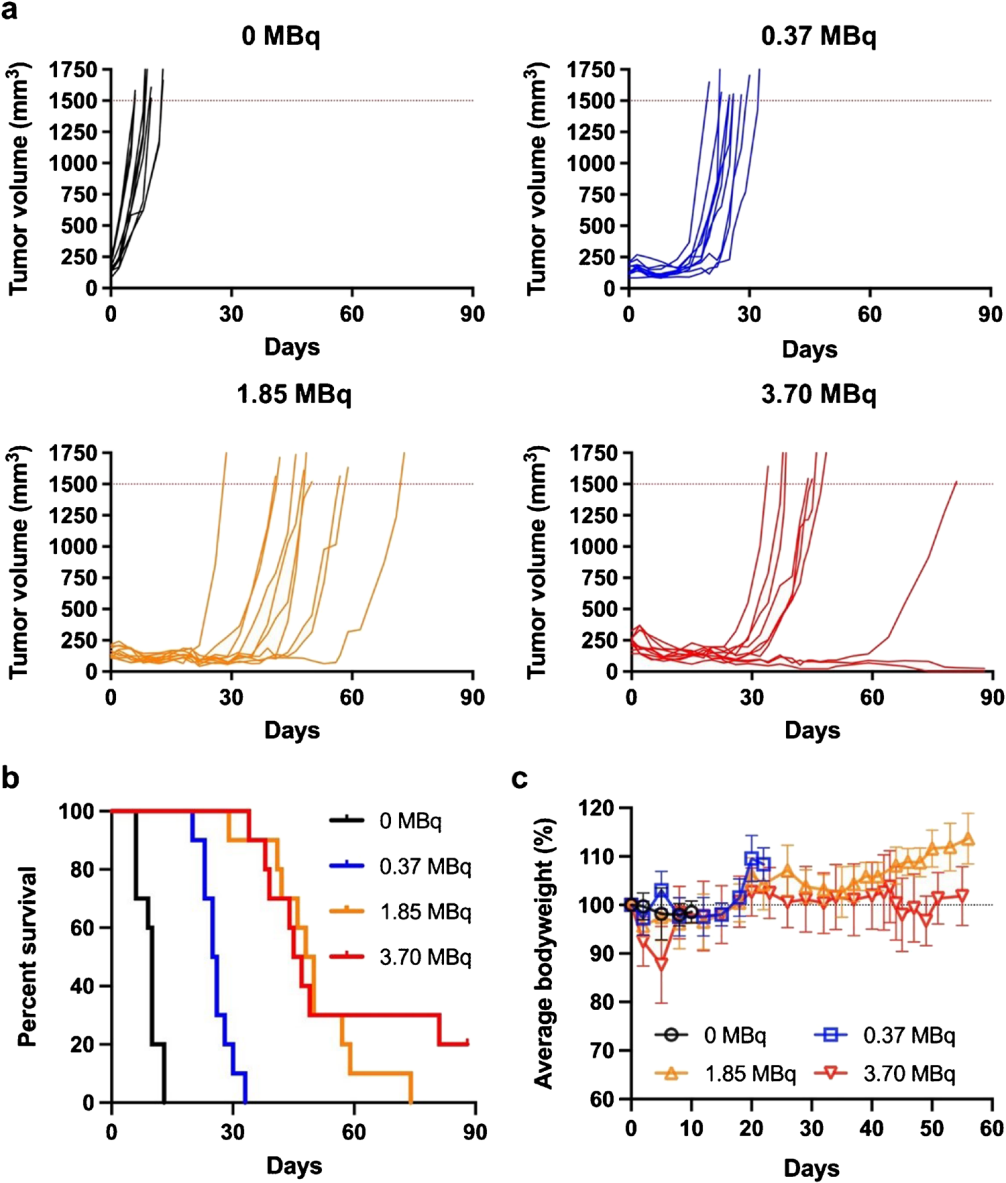
Therapeutic outcomes of [^212^Pb]Pb-PSC-PEG_2_-TOC therapy in AR42J tumor-bearing nude mice. [^212^Pb]Pb-PSC-PEG_2_-TOC therapy was initiated when the average tumor size became around 150 mm3. 0.37 MBq, 1.85 MBq, and 3.70 MBq of [^212^Pb]Pb-PSC-PEG_2_-TOC were administered via tail vein with co-administration of DL-lysine (400 mg/kg) to reduce the renal uptake of the radiotherapeutic. The therapeutic outcomes of [^212^Pb]Pb-PSC-PEG_2_-TOC were monitored by **a** individual tumor growth, **b** survival, and **c** bodyweight change over time. The median survival endpoints were 10, 26, 49, and 46 days for the control, 0.37 MBq, 1.85 MBq, and 3.70 MBq-treated groups, respectively (*n*=10 for each group)

^212^Pb therapy studies were designed based on the favorable biodistribution profile and imaging of [^203^Pb]Pb-PSC-PEG_2_-TOC. Mice bearing AR42J tumor xenografts were treated with escalating radioactivity doses of [^212^Pb]Pb-PSC-PEG_2_-TOC (i.e., 0.37 MBq, 1.85 MBq, and 3.70 MBq), which were co-administered with DL-lysine (400 mg/kg). Tumor growth was significantly suppressed and overall survival was improved in the ^212^Pb-treated groups compared to the untreated group, with a dose-dependent effect observed up to 1.85 MBq of [^212^Pb]Pb-PSC-PEG_2_-TOC (Fig. 6). The administration of 3.70 MBq of [^212^Pb]Pb-PSC-PEG_2_-TOC elicited two presumable cases of complete response by the end of the 90-day study, which was not achieved with the lower administered activities (Fig. 6a). However, the median survival was not significantly different compared to the 1.85 MBq-treated group (49 days for the 1.85 MBq-treated vs. 46 days for the 3.70 MBq-treated; Fig. 6b). 1.85 MBq of [^212^Pb]Pb-PSC-PEG_2_-TOC was well tolerated and did not cause significant bodyweight loss in the treated mice (Fig. 6c). On the other hand, the administration of 3.70 MBq of [^212^Pb]Pb-PSC-PEG_2_-TOC resulted in an average initial weight loss of about 13% (at day 5), which appears to be attributed to the administered radiopharmaceutical. Importantly however, the bodyweight of the mice was fully recovered within 8 days, suggesting that the effect is reversible. The dosimetry analysis estimated that the administration of 0.37 MBq, 1.85 MBq, and 3.70 MBq of [^212^Pb]Pb-PSC-PEG_2_-TOC delivered 3.2 Gy, 16.0 Gy, and 32.0 Gy of tumor doses, respectively, and 1.2 Gy, 6.0 Gy, and 12.0 Gy of kidney doses, respectively (Table 2).

**Fig. 7:**
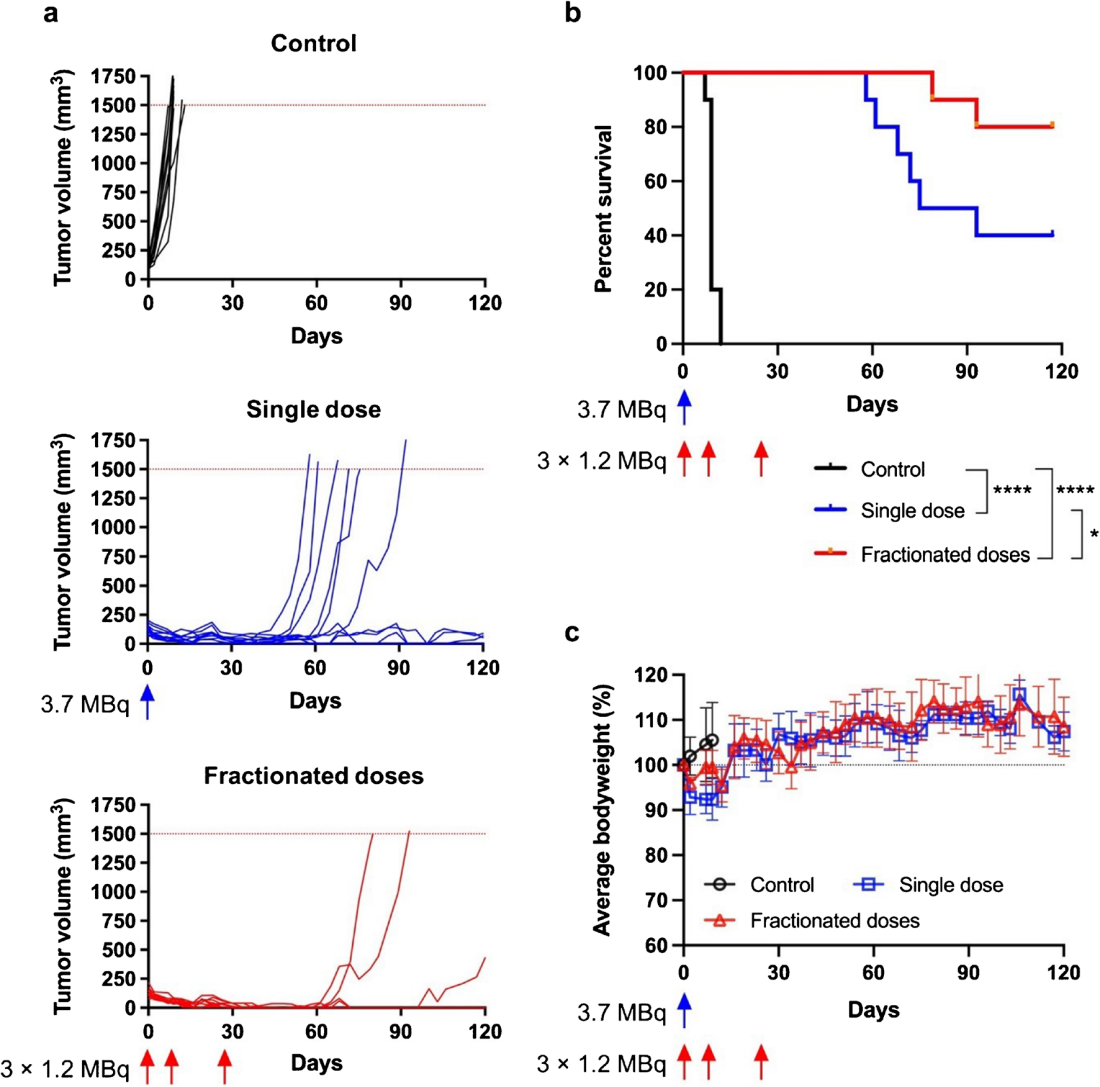
Therapeutic outcomes of [^212^Pb]Pb-PSC-PEG_2_-TOC in mice bearing AR42J tumor following administrations of single dose vs. three fractionated doses. Female nude mice bearing AR42J tumor xenograft (with mean tumor size of 123–137 mm3) were administered 0 MBq, 3.7 MBq (day 0), or 3 × 1.2 MBq (days 0, 9, and 28) of [^212^Pb]Pb-PSC-PEG_2_-TOC. The treatment intervals were not perfectly optimized due to a logistic issue of the ^224^Ra/^212^Pb generators during the COVID-19 pandemic. Results are presented for **a** tumor growth in each individual animal (*n*=10 per group); **b** Kaplan-Meier overall survival curves (*P<0.05; ****P<0.0001); and **c** mean bodyweight changes of treated mice and control mice

Another therapy study was conducted to compare the efficacy for a single (large) administered radioactivity dose with multiple fractionated doses of [^212^Pb]Pb-PSC-PEG_2_-TOC. The control mice reached the endpoint (tumor volume > 1500 mm^3^) within 12 days, with a median survival of 9 days (Fig. 7a, b). In this case, a significant inhibition of tumor growth was observed in the mice treated with a single dose of 3.70 MBq of [^212^Pb]Pb-PSC-PEG_2_-TOC, resulting in an improved median survival of 84 days and 40% complete responses were observed at the predetermined termination of the study (120 days) (Fig. 7a, b; *P*<0.0001 vs. control). Interestingly, a more robust therapeutic response was observed when [^212^Pb]Pb-PSC-PEG_2_-TOC was administered over three 1.2 MBq fractions (Fig. 7a), leading to an 80% survival rate at 120 days and 70% complete responses (Fig. 7b, *P*<0.05 vs. single-dose). Bodyweight losses were reversible in both treatment cohorts, indicating that 3.70 MBq of [^212^Pb]Pb-PSC-PEG_2_-TOC was well tolerated following either a single or fractionated dose regimen (Fig. 7c).

Histopathological changes at the conclusion of treatment with the single dose or with fractionated doses were scored into 5 categories, where score 0 represents the absence of toxicity and score 4 represents very severe toxicity (Fig. S4). For mice that received single dose of 3.7 MBq of [^212^Pb]Pb-PSC-PEG_2_-TOC (*n*=4), the occurrence of tubulointerstitial inflammation was mild (1–10%). Three out 4 mice showed absent or mild glomeruli injury. There was wide distribution of tubular injury among the 4 mice with 1 mouse in each category, suggesting potential individual variance. For the 8 mice that received 3 cycles of 1.2 MBq [^212^Pb]Pb-PSC-PEG_2_-TOC, 7/8 mice showed mild tubulointerstitial inflammation. Similar glomeruli injury score was found in both cohorts, except that one very severe scoring (>31%) was found in mice treated with fractionated [^212^Pb]Pb-PSC-PEG_2_-TOC. Moderate (11–25%) to severe (26–50%) occurrence of tubular injury was found in all treated animals at the conclusion of the study on day 120. As all control mice were euthanized before conclusion of the study due to aggressive tumor burden, no direct comparison with control cohort was possible.

## Discussion

In this investigation, synthetic design strategies were employed to modify the structure of an SSTR2-targeted peptide based on TOC to allow for the inclusion of Pb isotopes and to determine if these structural modifications could improve key performance metrics (e.g., in vitro binding affinity and internalization; in vivo tumor targeting and renal clearance rate), with a goal of enhancing the overall efficacy and tolerability of the radiopharmaceutical.

In previous studies, we had aimed at similar enhancements to a peptide construct targeted to the melanocortin receptor subtype 1 (MC1R) in metastatic melanoma. In this work, we examined a similar set of structural modifications beginning with analogs of the alpha-melanocyte stimulating hormone (a-MSH). A key structural modification that significantly enhanced the peptide performance in these earlier studies was the use of a triazole linkage for cyclization of the peptide. As we described earlier, the triazole linkage is obtained in a facile fashion via the Cu-catalyzed azide-alkyne cycloaddition (aka “click” chemical reactions) [26]. For the a-MSH analogs produced in the previous investigation, the fused triazole cyclization approach was shown to improve binding affinity and in vivo stability of the peptide in melanoma models. However, in the current investigation of TOC derivatives presented here, we found that the same click chemistry cyclization strategy almost completely abolished the binding of the RPDC to SSTR2. Here, results suggest that the binding of the SSTR2-targeted peptides to the receptor is influenced not only by the amino acid sequence, but also by the conformations of the amino acid sequence that is sensitive to cyclization structure, and that the fused triazole linkage disrupts the conformation in a way that inhibits receptor binding to SSTR2. These results highlight the difficulty in predicting the ultimate RPDC performance based on similar modifications made to other peptide sequences as has been reported previously [37, 38].

Insertion of PEG linkers has been explored as a means to manipulate the in vivo PK of various therapeutics [27]. Investigations in the use of PEG linker insertions for RPDCs have shown mixed results in terms of improvements to PK properties. For example, while certain studies identified that improved PK properties could be imparted by insertions or alterations in the lengths of PEG linkers [39, 40], other studies showed that PEG insertions made no difference in the ultimate performance of the RPDC [41]. In our study, we tested multiple lengths of PEG linkers and found that a PEG_2_ linker, inserted between the chelator and the peptide backbone, improved receptor-binding properties — presumably by minimizing the potential steric hindrances of the chelator-metal complex on SSTR2 binding without disrupting the conformation of the amino acids in the peptide backbone.

In the vivo setting, [^203^Pb]Pb-PSC-PEG_2_-TOC significantly improved the tumor-targeting properties (i.e., receptor binding, tumor accumulation and retention), compared to [^203^Pb]Pb-DOTATOC. In addition, the [^203^Pb]Pb-PSC-PEG_2_-TOC radiopharmaceutical exhibited faster renal clearance than the [^203^Pb]Pb-DOTATOC counterpart. Interestingly, although PSC-PEG_2_-TOC and PSCTOC (both unlabeled) had comparable IC_50_ receptor specific binding affinities, the cellular uptake of [^203^Pb]Pb-PSC-PEG_2_-TOC was significantly higher than [^203^Pb]Pb-PSCTOC. We attribute the improved cellular uptake of [^203^Pb]Pb-PSC-PEG_2_-TOC compared to [^203^Pb]Pb-PSCTOC to a decrease in the interference of the chelator-^203^Pb complex with SSTR2 binding due to the PEG_2_ linker insertion, which in turn leads to improved binding affinity to SSTR2. The fast renal kinetics of the PEG linker-inserted radiopeptide possibly results from increased hydrophilicity of the radiopeptide imparted by the PEG_2_ [39, 42, 43]. The biodistribution and the SPECT/CT imaging displayed faster renal clearance of [^203^Pb]Pb-PSC-PEG_2_-TOC without compromising tumor targeting, which significantly improved the therapeutic index of the therapy. PSC-PEG_2_-TOC also exhibited an excellent labeling yield and robust radiochemical stability (as well as metabolic stability) in water and human serum for ^203^Pb (Fig. S1 and Fig. S2). In our recently published study [44], we reported the favorable labeling and chelation properties of the PSC-conjugates (i.e., PSC-PEG_2_-TOC) for ^212^Pb as well as ^212^Bi, in comparison to DOTA-conjugated peptides (i.e., DOTA-PEG_2_-TOC and DOTATATE). High specific activities (up to 120 MBq/nmol; >90% efficiency) of [^203^Pb]Pb-PSC-PEG_2_-TOC were achieved in the present study (Fig. S3). Successful production of [^203^Pb]Pb-PSC-PEG_2_-TOC with high yield for clinical use has been reported [45], and [^203/212^Pb]Pb-PSC-PEG_2_-TOC theranostics are currently under phase I/IIa investigation (NCT05636618).

In the first therapy study with the single-dose regimen, significant tumor growth inhibition was observed in all treated animals, despite the bulky initial tumor size. The average tumor sizes upon initiation of treatment were 161 ± 42, 145 ± 41, 153 ± 37, and 234 ± 49 mm^3^ in the animals treated with 0 MBq, 0.37 MBq, 1.85 MBq, and 3.70 MBq of [^212^Pb]Pb-PSC-PEG_2_-TOC, respectively. Dose-dependent tumor inhibition was observed in animals treated with up to 1.85 MBq of [^212^Pb]Pb-PSC-PEG_2_-TOC, but not with 3.70 MBq. The lack of further improvement in the 3.70 MBq treatment cohort might be attributable to the significantly larger initial tumor size in this cohort, despite observing two out of ten complete tumor remission in this cohort.

In the second experiment, single-dose treatment with 3.70 MBq of [^212^Pb]Pb-PSC-PEG_2_-TOC was initiated when the average tumor size was 130 mm^3^. This treatment resulted in a significant improvement in median survival of the mice (84 days) and a complete response rate (40%), suggesting that the initial tumor size can significantly affect therapy efficacy in preclinical studies. These findings may be particularly significant because the short ranges of alpha-particles, which are less than 88 mm in the case of ^212^Pb decay daughters (accounting for a few cell diameters) [20], result in highly non-homogenous dose deposit due to the large tumor size and intra-tumoral heterogeneity of receptor expression [46]. Further improved outcomes were observed from fractionated dosing of [^212^Pb]Pb-PSC-PEG_2_-TOC. Fractionated doses induce prolonged radiation delivery, which can result in a pro-apoptotic environment and more effective tumor cell killing [47]. Conversely, a single high dose can cause rapid tumor cell death, followed by repopulation of surviving cells, leading to less durable long-term effect. Additionally, fractionation can upregulate target receptor expression, thereby increasing the tumor control of subsequent therapies [48]. Another advantage of fractionation is its potential to minimize receptor saturation by the RPDC and enable more complete coverage of the tumor with heterogeneous receptor expression, reducing the bypassing effect of the therapeutic agent. Further studies are required to develop a more detailed understanding of the most effective treatment regimen in this regard.

A recent study conducted by Stallons and collaborators [49] demonstrated that 1,4,7,10-tetraazacyclododecane-1,4,7,10-tetraacetic acid amide (DOTAM)-conjugated TATE (DOTAMTATE) has potential for NET therapy with ^212^Pb in a preclinical model. In the study, therapy with [^212^Pb]Pb-DOTAMTATE showed dose-dependent tumor control and a survival benefit in the same animal model used in our study, indicating the potential of ^212^Pb for NET therapy. The most effective treatment regimen was observed with three cycles of 0.37 MBq [^212^Pb]Pb-DOTAMTATE given at 2-week intervals. This treatment resulted in approximately 50% complete responses and a median survival of about 76 days post-treatment. In that study, the therapeutic dose was not increased due to the high toxicity profile of [^212^Pb]Pb-DOTAMTATE. In a toxicity study with tumor-free athymic nude mice, a dose of 1.48 MBq of [^212^Pb]Pb-DOTAMTATE or higher resulted in a 100% death rate within 10 days. In contrast, our study shows that administering up to 3.70 MBq [^212^Pb]Pb-PSC-PEG_2_-TOC in mice, with three fractionated doses (i.e., 1.20 MBq × 3 cycles), results in 70% complete responses and an 80% survival rate at 120 days. Notably, our study differed in study design (we used AR42J-bearing mice administered with DL-lysine). Nonetheless, we observed no toxicity-related mouse deaths from the administration of up to 3.70 MBq single bolus injection of [^212^Pb]Pb-PSC-PEG_2_-TOC. These results highlight the need for more studies to develop a more detailed understanding of the therapeutic index of [^212^Pb]Pb-PSC-PEG_2_-TOC and to identify potential further improvements through multi-dosing strategies. Results presented here, together with recent clinical imaging of the [^203^Pb]Pb-PSC-PEG_2_-TOC [50], provide compelling evidence that [^203^Pb/^212^Pb]Pb-PSC-PEG_2_-TOC has prodigious potential for image-guided receptor-targeted alpha-particle therapy for SSTR2-expressing tumors.

## Conclusion

Preclinical studies described here suggest that PSC-PEG_2_-TOC has the potential to improve the efficacy of Pb-based a-particle therapy for SSTR2-expressing tumors with a significantly lower toxicity profile than previous SSTR2-targeted peptides. Thus, PSC-PEG_2_-TOC is a promising candidate for the treatment of NET patients who are naïve or refractory to b-PRRT. Upcoming toxicity evaluation and therapeutic studies in multi-dosing regimen will provide more definitive information regarding the potential of this radiopharmaceutical.

We expect that this type of structural modification strategy can also be applied to other peptide-based therapeutics. To this end, experiments are under way to explore the potential improvement of the current (i.e., [^177^Lu]Lu-DOTATATE) and other therapeutics by optimizing the structure for a specific radionuclide. The results presented here highlight the difficulty in predicting impact of molecular modifications of structural changes on the PK properties of radiopharmaceuticals and the impact of chelator modifications to the stability of daughter radionuclides at the chelator-radiometal coupling.

Our findings underscore the potential benefits of fractionated RPDC therapy in cancer treatment. Fractionated doses can create a favorable environment for tumor cell apoptosis, enhance tumor control through receptor upregulation, and minimize receptor saturation. These advantages suggest that fractionation using RPDC may hold promise in optimizing cancer treatment regimens. However, further research is needed to determine the most effective and tailored approaches for harnessing these benefits in clinical practice.

### Supplementary information

ESM 1 (DOCX 655 kb)
