# Supplementary material for: Structural modifications toward improved lead-203/lead-212 peptide-based image-guided alpha-particle radiopharmaceutical therapies for neuroendocrine tumors
Source: Eur J Nucl Med Mol Imaging. 2023 Nov 13;51(4):1147–62. doi: 10.1007/s00259-023-06494-9 (PMC10881741; doi:10.1007/s00259-023-06494-9)
Supplement: Supplementary file 1 — (DOCX 655 kb) [file 259_2023_6494_MOESM1_ESM.docx]

**SUPPLEMENTARY MATERIAL**

**Structural modifications toward improved lead-203/lead-212 peptide-based image-guided alpha-particle radiopharmaceutical therapies for neuroendocrine tumors**

Dongyoul Lee^1^, Mengshi Li^2^, Dijie Liu^2^, Nicholas J. Baumhover^2^, Edwin A. Sagastume^2^, Brenna M. Marks^2^, Prerna Rastogi^3^, F. Christopher Pigge^4^, Yusuf Menda^5^, Frances L. Johnson^2^, and Michael K. Schultz*^2, 4, 5, 6^

^1^Department of Physics and Chemistry, Korea Military Academy, Seoul, Republic of Korea; ^2^Perspective Therapeutics, Inc., Coralville, IA, USA; ^3^Department of Pathology,

The University of Iowa Hospitals and Clinics, Iowa City, IA, USA; ^4^Department of Chemistry, The University of Iowa, Iowa City, IA, USA; ^5^Department of Radiology, The University of Iowa Hospitals and Clinics, Iowa City, IA, USA; ^6^Department of Radiation Oncology, The University of Iowa Hospitals and Clinics, Iowa City, IA, USA.

*Corresponding Author: Michael K. Schultz PhD, Associate Professor Emeritus; Departments of Radiology, Radiation Oncology, and Chemistry, The University of Iowa, ML B180 FRRBP, 500 Newton Road, Iowa City, IA 52240, Phone +1 (319) 335-8017 Email michael-schultz@uiowa.edu

**Methods**

**Peptide synthesis**

DOTATOC, DOTA-click-TOC, PSCTOC, PSC-PEG_2_-TOC, and PSC-PEG_4_-TOC were synthesized using standard fluorenylmethyloxycarbonyl (Fmoc)-based solid phase peptide synthesis. The linear peptides, TOC and click-TOC, were first synthesized at 0.1 mmol scale on 2-chlorotrityl (2-CT) resin following standard Fmoc procedures using an automated peptide synthesizer (AAPPTEC Apex 396). For click-TOC, propargylglycine (Pra) and azido-lysine (Lys(N_3_)) were substituted for two cysteines of TOC. The N-termini of the linear peptides were deprotected by 25% piperidine (PIP) at the end of automated synthesis.

The copper (Cu)-catalyzed cyclization for click-TOC was conducted before the chelator conjugation step to minimize the potential chelation of Cu, which was used in the reaction for the alkyne-azide cycloaddition while the cysteine-mediated cyclization for TOC was conducted after the chelator conjugation. For the click chemistry cyclization of click-TOC, the linear click-TOC on the resin was added with 1 equivalence (1 equiv.) of Cu(Ι) bromide, 3 equiv. of sodium ascorbate, 10 equiv. of N,N-diisopropylethylamine (DIPEA), 10 equiv. of 2,6-lutidine relative to the amount of peptide in dimethylformamide (DMF) and reacted with mild stirring (150 rpm) at 37 °C overnight.

Manual addition of the PEG linker (PEG_2_ or PEG_4_) was performed for the PEGylated peptides, PSC-PEG_2_-TOC or PSC-PEG_4_-TOC. The peptides on the resin were suspended with DMF, and 5 equiv. of Fmoc-NH-PEG_2_/PEG_4_-propionic acid (purchased from AAPPTEC), 2-(7-aza-1H-benzotriazole-1-yl)-1,1,3,3-tetramethyluronium hexafluorophosphate (HATU), and 1-hydroxybenzotriazole (HOBt), and 10 equiv. of DIPEA were added and reacted while being mixed at 37 °C for 2 h. The Fmoc-protection groups on the N-termini of the peptide-resins were then manually deprotected by 25% PIP in DMF with mild mixing at 25°C for 10 min and washed with DMF/dichloromethane (DCM)/methanol. The process was repeated to increase the yield.

The peptides with open N-termini on the resins were then resuspended in DMF, and 5 equiv. of either DOTA-tris(tert-butyl ester) or PSC-bis(tert-butyl ester), HATU, and HOBt, and 10 equiv. of DIPEA were added and reacted at 37 °C while being mixed overnight. The success of each step of coupling/deprotection was verified by the Kaiser test [1], and the process was repeated until successful.

The linear TOC-based peptides (with cysteines) conjugated with either DOTA or PSC were then cyclized by iodine oxidation. Iodine (I_2_; 20 equiv.) was dissolved in 6 ml DMF and added to the peptides on the resin, and a reaction was allowed for 3 h for trityl deprotection from cysteine and concomitant promotion of disulfide formation *via* oxidation.

The resin and protecting groups were cleaved from the cyclized peptides by adding 3 mL cleavage cocktail (93% trifluoroacetic acid, 3% triisopropylsilane, 4% water) for 2 h at room temperature, followed by ether precipitation on ice for at least 4 h. The crude peptides were then purified by semi-preparative HPLC with a C-18 column (Vydac 10 × 250 mm, 10 μm; Grace, Deerfield, IL). The collected samples were concentrated by rotary evaporation, and lyophilized. The purified peptides were characterized by a mass spectrometer and had purity levels of >97%.

**^212^Pb dose calibration**

To minimize the uncertainty in the measurement of ^212^Pb radiopharmaceutical doses, we obtained a liquid ^232^U source (hermetically-sealed ampoule; Eckert & Ziegler; 3.391 kBq) which maintained radioactive equilibrium of ^212^Pb with its decay daughters. On the day of an experiment, we determined the counting efficiency (62.0–63.7%) of ^212^Pb-specific peak (239 keV) by counting the radioactivity for 2 mins using a sodium iodide scintillation [NaI(Tl)] detector, taking into account the intensity (43.6%) of the 239 keV photons. ^212^Pb samples were then measured in the NaI(Tl) detector for counts per second (CPS); the CPS was converted to ^212^Pb activity taking into account the pre-determined counting efficiency and the intensity of the 239 keV photons from ^212^Pb decays. These calculated values were in good agreement with theoretical values (<10%).

**^203^Pb/^212^Pb radiolabeling, HPLC separation, and dose preparation**

^203^Pb (obtained from Lantheus Medical Imaging, North Billerica, MA) or ^212^Pb (eluted from a radium-224 (^224^Ra)/^212^Pb generator, Oak Ridge National Laboratories, Oak Ridge, TN) was purified and concentrated using the Pb-specific extraction resin (Pb-Resin™, Eichrom Technologies, Lisle, IL) and eluted with pH=6 sodium acetate (NaOAc) buffer. The isolated Pb isotopes (pH=6 in NaOAc buffer; 1 mL) were then added to the pH=4 NaOAc buffer (145 µL; final pH= 5.3–5.5) containing peptides. The reactions were allowed to proceed at 85°C for 20–30 min. For the ^212^Pb reactions, ascorbic acid (1 mg/ml) was added to protect the oxidation and degradation of the peptide. The product was purified by a C-18 reverse-phase cartridge (Strata-X Solid Phase Extraction, SPE; Phenomenex, Torrance, CA) and diluted in saline (<5% ethanol; 1 mg/ml ascorbic acid). In the case of ^212^Pb doses, DL-lysine was added to reduce the renal uptake of the radiotherapeutics.

For cellular uptake assay of ^203^Pb-labeled peptides, 37 MBq of ^203^Pb were labeled with 10 nmol of DOTATOC, PSCTOC, and PSC-PEG_2_-TOC. The labeled peptides were separated from the unlabeled by a HPLC system (Agilent 1200 Series) based on differential retention of the labeled and the unlabeled peptides by the previously developed method [2]. Briefly, the retention time of ^203^Pb-labeled and unlabeled peptides were first determined using the HPLC system. The run was initiated with 5 min equilibrium in 4% acetonitrile (ACN) in 20 mM HCl, and the organic phase increased to 16% in 1 min, followed by a linear 16–26% ACN gradient over 20 minutes with a 1ml/min flow rate (at 37 °C on a Vydac 218TP C18 column; 4.6×150 mm, 5 µm). The differential retention (1.5–3 min; labeled vs. unlabeled) allowed manual collection of the ^203^Pb-labeled peptides, which resulted in high specific activities of the radiotracers. The HPLC-separated products were purified by C-18 SPE column.

**Analysis of radiochemical yield for ^203^Pb/^212^Pb radiolabeling**

DOTATOC and the PSC-conjugated peptides were radiolabeled with ^203^Pb and ^212^Pb. 18.5 MBq of ^203^Pb or 14.1 MBq of ^212^Pb was reacted with 10 nmol peptides in 0.5 M NaOAc buffer (pH=5.4; 1 ml reaction volume). The reaction was conducted at various temperatures (25, 50, or 85 °C) and reaction time (10, 20, or 30 min) for the ^203^Pb labeling. DOTATOC and PSCTOC were selected for the ^212^Pb labeling performance test, and the reaction was conducted at a fixed temperature (85 °C) with increasing reaction time (up to 30 min). After the reaction, a sample of the resultant mixture (2 μl) was spotted on pre-dried instant thin layer chromatography (iTLC) strips and developed by 10 mM diethylenetriaminepentaacetic acid (DTPA) in 0.1 M NaOAc buffer. The strips were then cut by half and the radioactivity of each portion (the top for free Pb; the bottom for Pb-labeled peptides) of each iTLC strip was measured by the isotope-specific gamma peaks (^203^Pb, 279 keV; ^212^Pb, 239 keV) using the NaI(Tl) detector.

Stability of [^203^Pb]Pb-PSC-PEG_2_-TOC in water and human serum

PSC-PEG_2_-TOC was characterized for stability in water and human serum. The peptide was radiolabeled with 50 MBq of ^203^Pb and purified by a C-18 process. 9 MBq of the purified radiopeptide was added into 3 ml water or normal human serum (S1-100ML; Sigma-Aldrich) and incubated at 37 °C for up to 24 h. After incubation, the serum samples with [^203^Pb]Pb-PSC-PEG_2_-TOC were transferred to Amicon Ultra Centrifugal Filter (3K; Millipore) and centrifuged by a Beckman Coulter Avanti J-25I centrifuge. The penetrate by centrifugation (serum samples) or the samples in water were analyzed by a radio-HPLC system (Agilent 1200 Series connected with an IN/US β-RAM Model 4 radio-detector) to monitor the degree of peptide degradation.

**Radiolabeling of [^203^Pb]Pb-PSC-PEG_2_-TOC for high molar activity**

^203^Pb was radiolabeled with PSC-PEG_2_-TOC to achieve clinically relevant molar activity of 90 MBq/nmol or 120 MBq/nmol. For reference, DOTATOC was also radiolabeled to 90 MBq/nmol. The reaction was conducted in 0.5 M NaOAc buffer (pH=5.4, 1–2 ml reaction volume) at 85 ºC for 30 min; 2 µl of reaction product containing ^203^Pb-labeled peptide was spotted on an iTLC strip. The strip was developed in a mobile phase (0.2 M NaOAc with 20 mM ethylenediaminetetraacetic acid, EDTA) and imaged with a phosphor imager (Typhoon FLA7000). The strip was cut in half and the radioactivity of each side of the strip was measured by the NaI(Tl) detector by the ^203^Pb-specific gamma peak (279 keV) to determine the radiolabeling efficiency.

**Tumor and kidney dosimetry**

Particle and Heavy Ion Transport code System (PHITS) [3] was used for dose deposit calculations in the kidneys and tumor (**Fig. 5a**). The DigiMouse voxel phantom model [4] was employed for renal dose estimation. The voxel size of the phantom model was adjusted to match with the average kidney volume (288.7 ± 41.4 mg; 28 mice) of female athymic nude mice (8–10 weeks old). The elemental composition and the mass density of the kidneys were assumed to be identical to the human reference adult values obtained from the International Commission on Radiation Units and measurements (ICRU) report 46 [5]. For tumor dose estimation, a spherical volume was constructed based on a tumor mass of 200 mg. The elemental composition and the mass density of the tumor were adapted from available data (adenoid cystic carcinoma; 1.04 g/cm^3^) [6, 7]. At least 1 million particles were transported for Monte Carlo calculations, which reduced the statistical uncertainty to less than 1%. Time-activity curves of ^212^Pb-labeled peptides in tumors and kidneys of female nude mice were based on the biodistribution of ^203^Pb-labeled peptides (**Fig. 3**), considering the ^212^Pb physical decay. The clearance of the radiopeptides was assumed to follow single-exponential decay curves (**Fig. 5b/c**).

**Results**

**PSC-PEG_2_-TOC exhibited favorable labeling and stability properties**

A radiolabeling yield greater than 99% of ^203^Pb was observed in DOTATOC, PSCTOC, PSC-PEG_2_-TOC, and PSC-PEG_4_-TOC at all tested temperatures (**Fig. S1**). The labeling efficiencies of DOTA and PSC for ^203^Pb at 25 °C were as good as those with the higher temperatures tested (i.e., 50, 85 °C), suggesting practical advantages of room temperature radiolabeling for Pb isotopes using the chelators. PSC-PEG_2_-TOC was evaluated for the radiolabeling yield with clinically relevant high ^203^Pb activity (**Fig. S3**). The peptide showed high labeling yields (>92%) at activity levels of ^203^Pb as high as 1.8–3.6 GBq, which resulted in high specific activities of 90–120 MBq/nmol. This demonstrates that high-yield production of [^203^Pb]Pb-PSC-PEG_2_-TOC with high activity levels, clinically relevant for human imaging, can be achieved with a high specific activity. Furthermore, the stability of [^203^Pb]Pb-PSC-PEG2-TOC was confirmed after a 24-hour incubation in water and human serum at 37 °C as assessed by the radio-HPLC, demonstrating excellent radiochemical and metabolic stability (**Fig. S2**).

**Figures and Tables**


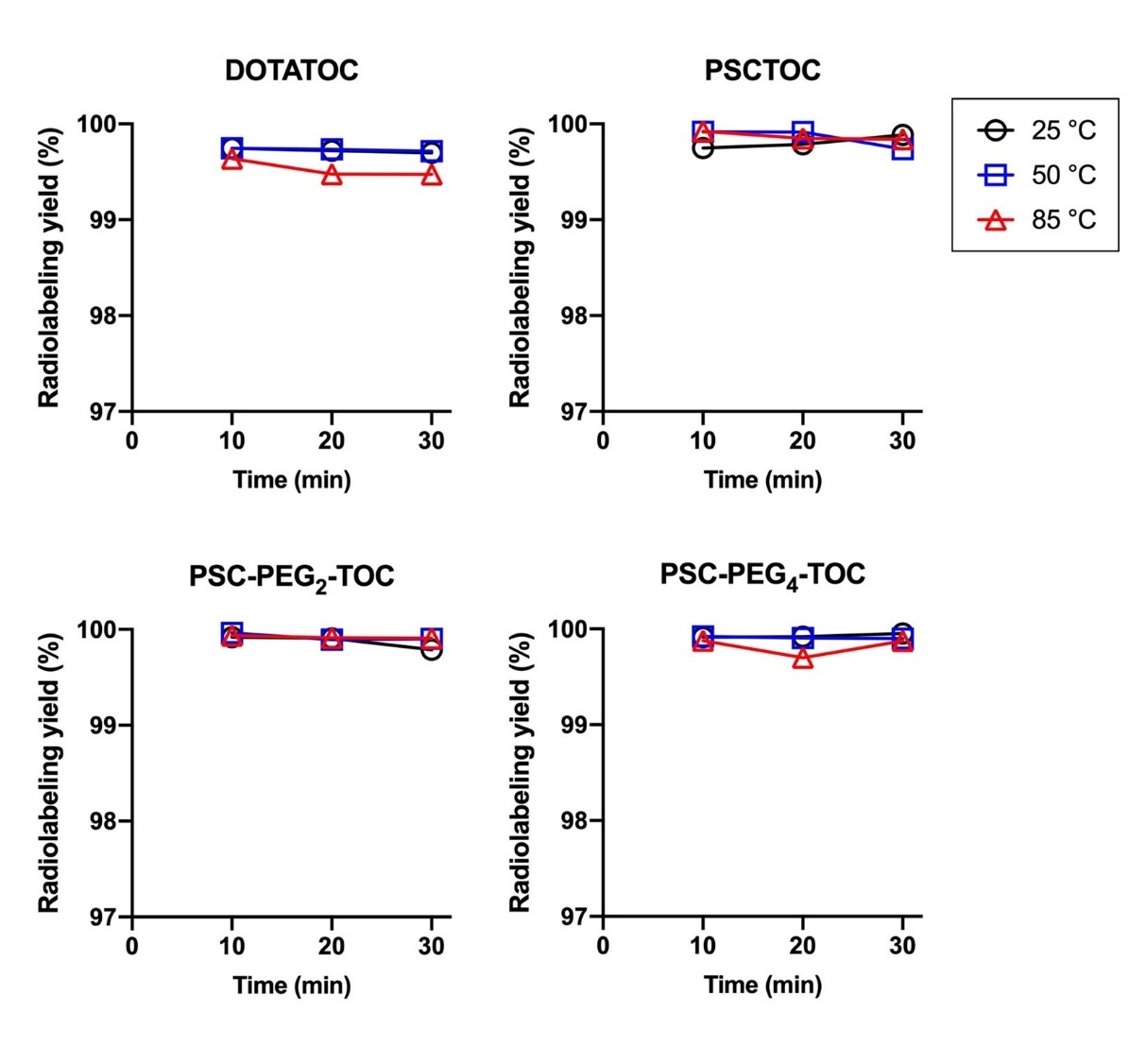


**Fig. S1** Radiolabeling efficiency of ^203^Pb for DOTATOC and the PSC-conjugated peptides. 18.5 MBq of ^203^Pb were reacted with 10 nmol peptides in 0.5 M NaOAc buffer (pH=5.4, 1 ml reaction volume) at various temperatures (25, 50, or 85°C) and reaction times (10, 20, or 30 min)

**
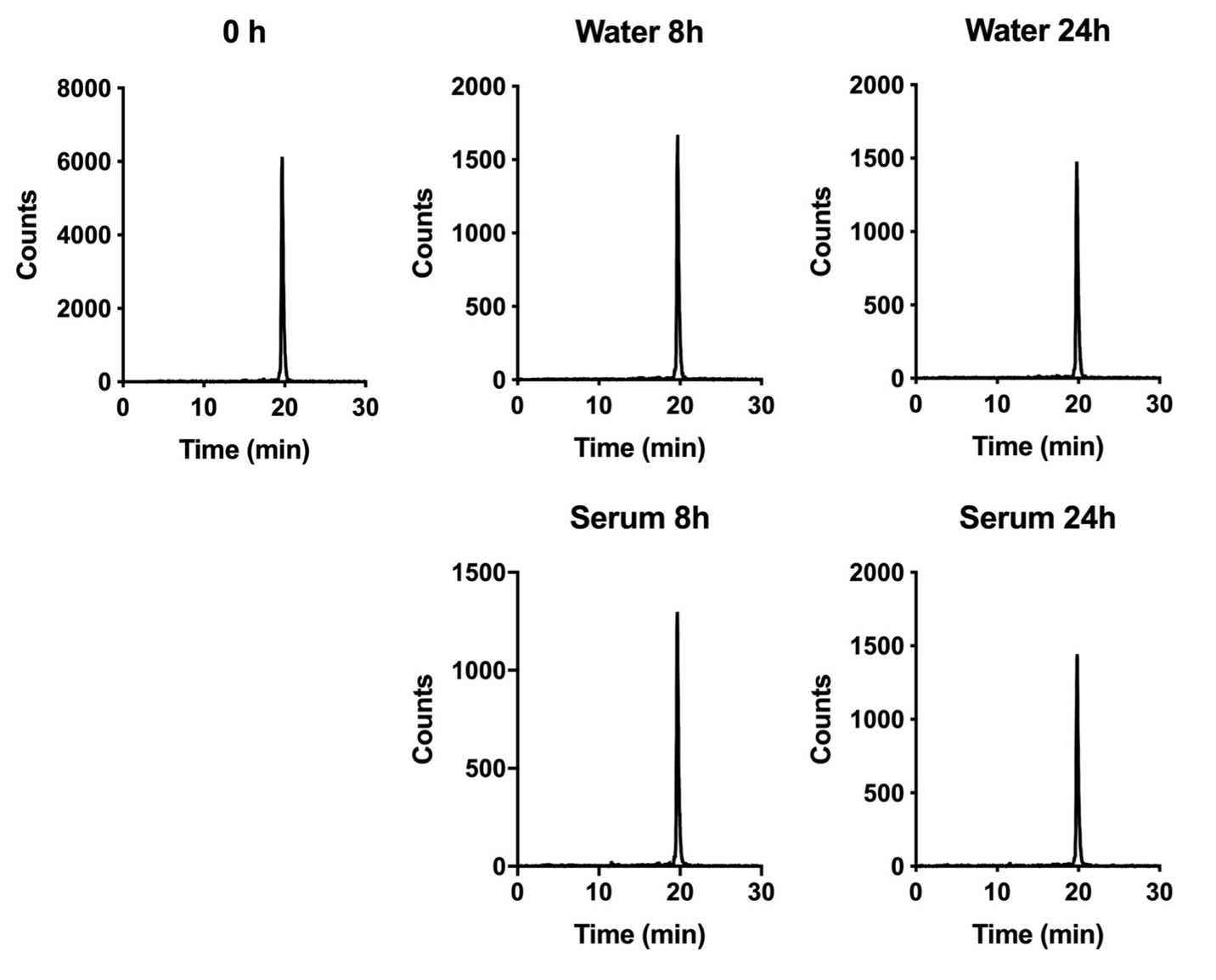
**

**Fig. S2** Radiochemical and structural stability of [^203^Pb]Pb-PSC-PEG_2_-TOC in water and human serum. PSC-PEG_2_-TOC was labeled with 50 MBq of ^203^Pb. After purification, 9 MBq of the purified radiopeptide were added to 3 mL of water or human serum and incubated at 37 °C for up to 24 h. Peptide degradation was monitored by radio-HPLC (Agilent 1200 Series connected with an IN/US β-RAM Model 4 radio-detector) at 8 h and 24 h post-incubation


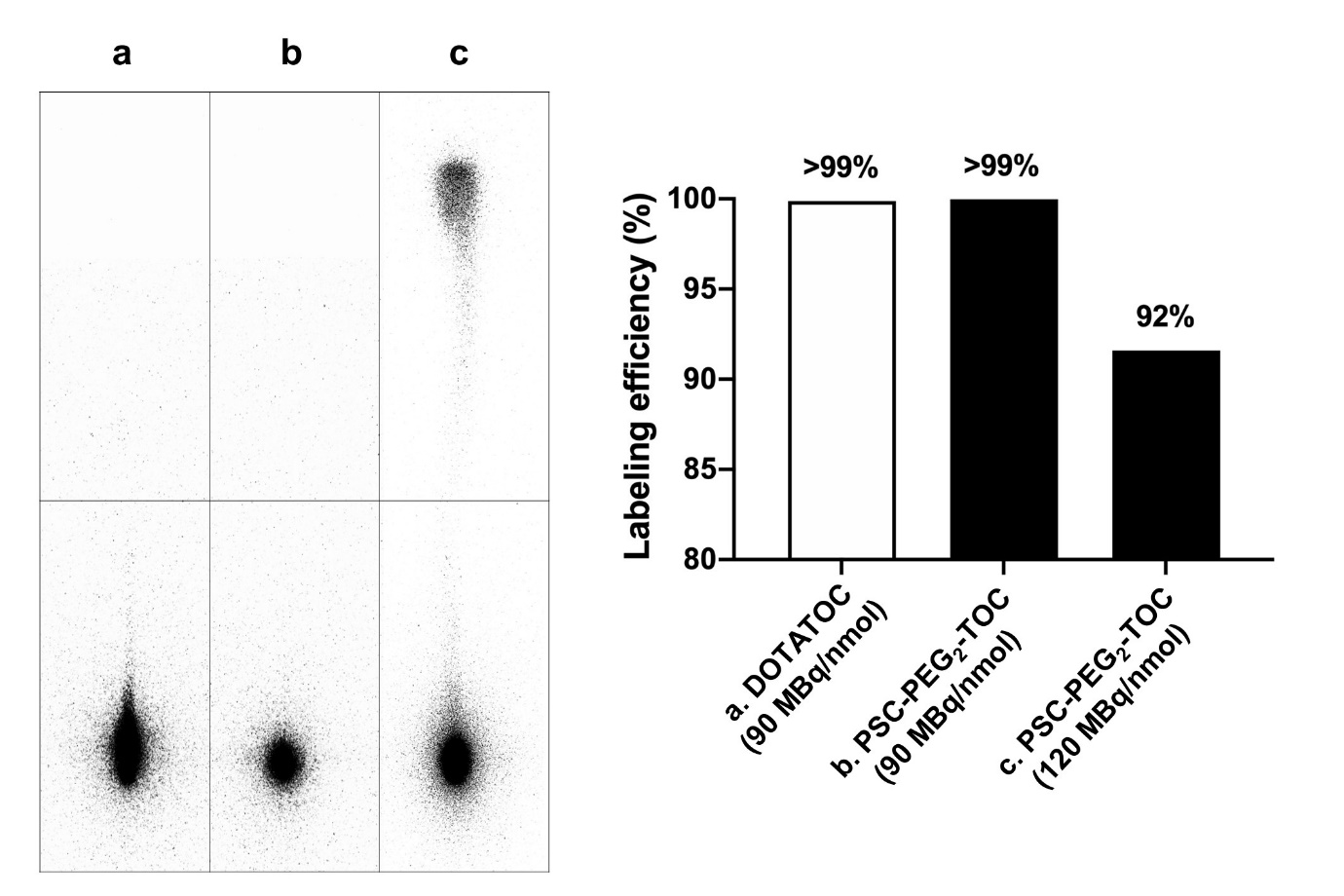


**Fig. S3** Clinically relevant high molar activity ^203^Pb radiolabeling of PSC-PEG_2_-TOC. **(a)** 90 MBq/nmol of DOTATOC as a reference; **(b)** 90 MBq/nmol of PSC-PEG_2_-TOC; **(c)** 120 MBq/nmol of PSC-PEG_2_-TOC. Experiments were carried out in 0.5 M NaOAc buffer (pH=5.4, 1–2 ml reaction volume) at 85 ºC for 30 min

| **Scoring:** | **Tubulointerstitial Inflammation** | **Glomeruli Injury** | **Tubular Injury** |
| --- | --- | --- | --- |
| 0- Absent | 0% | 0% | 0% |
| 1- Mild | 1-10% | 1-10% | 1-10% |
| 2- Moderate | 11-25% | 11-20% | 11-25% |
| 3- Severe | 26-50% | 21-30% | 26-50% |
| 4- Very Severe | > 50% | > 31% | > 50% |
| Tubular Changes: stained bodies of various sizes, vacuolization, loss of epithelial cells nuclei, dark acidophilic cytoplasm, loss of tubular epithelial cells into tubular lumen, and acellular sections of tubules. | | | |
| Glomerular changes: glomeruli with any degree of sclerosis or collapse and thrombonecrotic lesions. | | | |

**
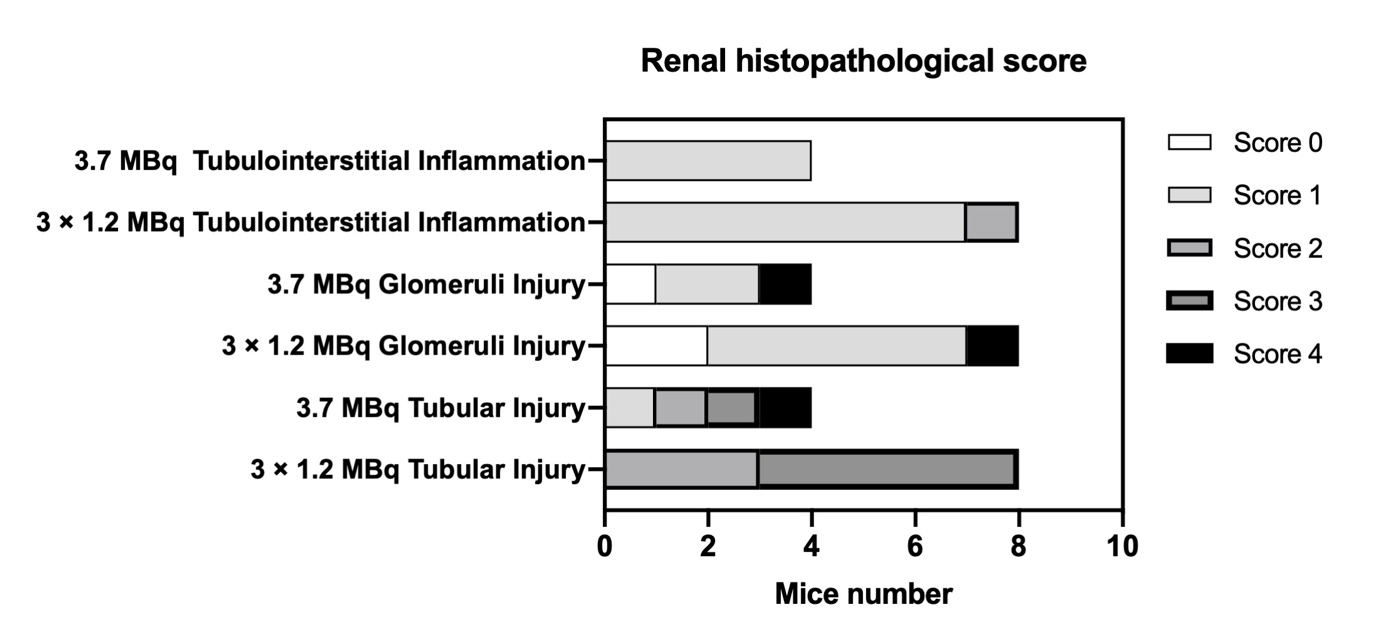
**

**Fig. S4** Histopathological scoring of renal toxicity resulting from single dose vs. three fractionated doses of [^212^Pb]Pb-PSC-PEG_2_-TOC in AR42J tumor bearing mice at conclusion of the study on 120 days post administration

**Table S1** Biodistribution of [^203^Pb]Pb-DOTATOC, [^203^Pb]Pb-PSCTOC, and [^203^Pb]Pb-PSC-PEG_2_-TOC at 1 h post-administration (%ID/g ± SD)

| Tissue | [^203^Pb]Pb-DOTATOC | [^203^Pb]Pb-PSCTOC | [^203^Pb]Pb-PSC-PEG_2_-TOC | |
| --- | --- | --- | --- | --- |
| Blood | 0.29 ± 0.02 | 0.53 ± 0.06 | 0.36 ± 0.10 | |
| Heart | 0.18 ± 0.02 | 0.32 ± 0.02 | 0.20 ± 0.05 |  |
| Liver | 0.28 ± 0.05 | 0.44 ± 0.02 | 0.36 ± 0.05 |  |
| Kidneys | 12.15 ± 2.14 | 13.61 ± 0.75 | 20.64 ± 2.75 |  |
| Lung | 0.91 ± 0.41 | 5.06 ± 0.74 | 3.58 ± 1.64 |  |
| Tumor | 4.60 ± 0.18 | 19.14 ± 3.31 | 28.18 ± 8.63 |  |
| Spleen | 0.27 ± 0.05 | 0.70 ± 0.13 | 1.21 ± 0.17 |  |
| Muscle | 0.10 ± 0.03 | 0.25 ± 0.06 | 0.29 ± 0.27 |  |
| Brain | 0.03 ± 0.01 | 0.16 ± 0.16 | 0.04 ± 0.01 |  |
| Small  intestine | 1.91 ± 0.80 | 1.38 ± 0.07 | 1.34 ± 0.35 |  |
| Large intestine | 0.32 ± 0.12 | 0.77 ± 0.04 | 1.24 ± 0.12 |  |
| Tumor-to-normal tissue ratio | | | |  |
| Tumor/blood | 15.86 | 36.11 | 78.28 |  |
| Tumor/liver | 16.43 | 43.50 | 78.28 |  |
| Tumor/kidneys | 0.38 | 1.41 | 1.37 |  |
| Tumor/lung | 5.05 | 3.78 | 7.87 |  |
| Tumor/muscle | 46.00 | 76.56 | 97.17 |  |

**Table S2** Biodistribution of [^203^Pb]Pb-DOTATOC, [^203^Pb]Pb-PSCTOC, and [^203^Pb]Pb-PSC-PEG_2_-TOC at 3 h post-administration (%ID/g ± SD)

| Tissue | [^203^Pb]Pb-DOTATOC | [^203^Pb]Pb-PSCTOC | [^203^Pb]Pb-PSC-PEG_2_-TOC | |
| --- | --- | --- | --- | --- |
| Blood | 0.02 ± 0.00 | 0.03 ± 0.01 | 0.05 ± 0.00 | |
| Heart | 0.07 ± 0.05 | 0.07 ± 0.00 | 0.08 ± 0.02 |  |
| Liver | 0.16 ± 0.02 | 0.19 ± 0.03 | 0.23 ± 0.02 |  |
| Kidneys | 14.60 ± 2.34 | 8.35 ± 1.33 | 15.79 ± 1.11 |  |
| Lung | 0.59 ± 0.23 | 1.09 ± 0.36 | 0.38 ± 0.07 |  |
| Tumor | 4.47 ± 0.75 | 16.20 ± 1.76 | 22.79 ± 2.80 |  |
| Spleen | 0.19 ± 0.04 | 0.35 ± 0.12 | 0.54 ± 0.29 |  |
| Muscle | 0.04 ± 0.01 | 0.05 ± 0.03 | 0.03 ± 0.01 |  |
| Brain | 0.01 ± 0.00 | 0.01 ± 0.00 | 0.02 ± 0.00 |  |
| Small  intestine | 1.78 ± 2.19 | 2.85 ± 2.25 | 1.85 ± 0.22 |  |
| Large intestine | 1.11 ± 0.74 | 3.10 ± 0.12 | 6.32 ± 0.68 |  |
| Tumor-to-normal tissue ratio | | | |  |
| Tumor/blood | 230.00 | 540.00 | 455.80 |  |
| Tumor/liver | 28.75 | 85.26 | 99.09 |  |
| Tumor/kidneys | 0.32 | 1.94 | 1.44 |  |
| Tumor/lung | 7.80 | 14.86 | 59.97 |  |
| Tumor/muscle | 115.00 | 324.00 | 759.67 |  |

**Table S3** Biodistribution of [^203^Pb]Pb-DOTATOC, [^203^Pb]Pb-PSCTOC, and [^203^Pb]Pb-PSC-PEG_2_-TOC at 24 h post-administration (%ID/g ± SD)

| Tissue | [^203^Pb]Pb-DOTATOC | [^203^Pb]Pb-PSCTOC | [^203^Pb]Pb-PSC-PEG_2_-TOC | |
| --- | --- | --- | --- | --- |
| Blood | 0.01 ± 0.00 | 0.01 ± 0.00 | 0.02 ± 0.00 | |
| Heart | 0.02 ± 0.01 | 0.05 ± 0.01 | 0.03 ± 0.01 |  |
| Liver | 0.08 ± 0.01 | 0.14 ± 0.00 | 0.08 ± 0.04 |  |
| Kidneys | 7.60 ± 0.91 | 5.59 ± 1.30 | 1.46 ± 0.38 |  |
| Lung | 0.27 ± 0.04 | 1.39 ± 0.27 | 1.46 ± 0.40 |  |
| Tumor | 2.44 ± 0.19 | 8.65 ± 0.70 | 12.21 ± 3.81 |  |
| Spleen | 0.10 ± 0.01 | 0.24 ± 0.03 | 0.28 ± 0.07 |  |
| Muscle | 0.02 ± 0.00 | 0.03 ± 0.01 | 0.02 ± 0.01 |  |
| Brain | 0.01 ± 0.00 | 0.02 ± 0.00 | 0.01 ± 0.00 |  |
| Small  intestine | 0.08 ± 0.06 | 0.12 ± 0.01 | 0.17 ± 0.01 |  |
| Large intestine | 0.17 ± 0.04 | 0.45 ± 0.03 | 0.83 ± 0.45 |  |
| Tumor-to-normal tissue ratio | | | |  |
| Tumor/blood | 460.00 | 865.00 | 610.50 |  |
| Tumor/liver | 57.50 | 61.79 | 152.63 |  |
| Tumor/kidneys | 0.61 | 1.55 | 8.36 |  |
| Tumor/lung | 17.04 | 6.22 | 8.36 |  |
| Tumor/muscle | 230.00 | 288.33 | 610.50 |  |

**Table S4** Biodistribution of [^203^Pb]Pb-PSC-PEG_2_-TOC with co-administration of DL-Lysine in AR42J tumor-bearing female nude mice at various points of time (1, 3, 6, and 24 h post administration). The data are presented as percent injected dose per gram of tissue (%ID/g ± SD, n=3 for each group)

| Tissue | 1 h | 3 h | 3 h  (No Lys)^a^ | 3 h  (Blocking)^b^ | 6 h | 24 h | |
| --- | --- | --- | --- | --- | --- | --- | --- |
| Blood | 0.36 ± 0.15 | 0.07 ± 0.02 | 0.07 ± 0.02 | 0.13 ± 0.02 | 0.05 ± 0.03 | 0.01 ± 0.00 | |
| Heart | 0.28 ± 0.08 | 0.15 ± 0.03 | 0.20 ± 0.13 | 0.07 ± 0.03 | 0.07 ± 0.05 | 0.03 ± 0.01 |  |
| Liver | 0.44 ± 0.08 | 0.25 ± 0.05 | 0.31 ± 0.03 | 0.30 ± 0.07 | 0.17 ± 0.02 | 0.06 ± 0.01 |  |
| Kidneys | 13.31 ± 3.80 | 9.84 ± 0.88 | 22.13 ± 1.92 | 40.90 ± 9.92 | 5.44 ± 0.63 | 1.25 ± 0.86 |  |
| Lung | 4.48 ± 4.47 | 4.76 ± 3.19 | 4.61 ± 3.75 | 0.26 ± 0.03 | 5.60 ± 1.27 | 1.11 ± 0.74 |  |
| Stomach | 1.46 ± 0.32 | 2.36 ± 0.44 | 0.95 ± 0.13 | 0.36 ± 0.15 | 0.78 ± 0.20 | 0.30 ± 0.12 |  |
| Pancreas | 5.04 ± 1.33 | 2.10 ± 0.68 | 2.29 ± 1.63 | 0.22 ± 0.04 | 0.75 ± 0.16 | 0.33 ± 0.08 |  |
| Tumor | 24.39 ± 1.85 | 22.95 ± 1.09 | 19.80 ± 1.61 | 2.41 ± 0.46 | 18.91 ± 1.86 | 7.44 ± 0.41 |  |
| Spleen | 1.04 ± 0.40 | 0.81 ± 0.30 | 0.97 ± 0.47 | 0.14 ± 0.07 | 0.73 ± 0.17 | 0.41 ± 0.28 |  |
| Muscle | 0.48 ± 0.26 | 0.03 ± 0.01 | 0.17 ± 0.10 | 0.06 ± 0.03 | 0.02 ± 0.02 | 0.01 ± 0.01 |  |
| Brain | 0.04 ± 0.01 | 0.03 ± 0.01 | 0.08 ± 0.06 | 0.03 ± 0.01 | 0.02 ± 0.00 | 0.01 ± 0.00 |  |
| Small intestine | 1.90 ± 0.74 | 2.37 ± 0.78 | 0.60 ± 0.11 | 0.81 ± 0.71 | 0.94 ± 0.32 | 0.15 ± 0.02 |  |
| Large intestine | 1.99 ± 0.31 | 3.97 ± 0.97 | 1.92 ± 0.56 | 0.99 ± 0.46 | 3.34 ± 0.63 | 1.01 ± 0.67 |  |
| Bone | 1.48 ± 0.34 | 0.48 ± 0.05 | 0.62 ± 0.24 | 0.16 ± 0.10 | 0.35 ± 0.06 | 0.16 ± 0.04 |  |
| Bone marrow | 2.42 ± 0.32 | 4.13 ± 1.55 | 4.17 ± 1.51 | 0.99 ± 0.42 | 1.69 ± 0.34 | 0.87 ± 0.60 |  |

^a^ At the 3 h post-administration, biodistribution of [^203^Pb]Pb-PSC-PEG_2_-TOC in the same animal model was assessed without co-administration of DL-Lysine.

^b^ Excessive amount (10 nmol) of unlabeled PSC-PEG_2_-TOC was co-administered to confirm the specificity of SSTR2 binding.

**References**

1. Kaiser E, Colescott RL, Bossinger CD, Cook PI. Color test for detection of free terminal amino groups in the solid-phase synthesis of peptides. Anal Biochem. 1970;34:595-8. https://doi:10.1016/0003-2697(70)90146-6.

2. Li M, Zhang X, Quinn TP, Lee D, Liu D, Kunkel F, et al. Automated cassette-based production of high specific activity (203/212)Pb peptide-based theranostic radiopharmaceuticals for image-guided radionuclide therapy for cancer. Appl Radiat Isot. 2017;127:52-60. https://doi:10.1016/j.apradiso.2017.05.006.

3. Sato T, Iwamoto Y, Hashimoto S, Ogawa T, Furuta T, Abe S-i, et al. Features of Particle and Heavy Ion Transport code System (PHITS) version 3.02. Journal of Nuclear Science and Technology. 2018;55:684-90. doi:10.1080/00223131.2017.1419890.

4. Dogdas B, Stout D, Chatziioannou AF, Leahy RM. Digimouse: a 3D whole body mouse atlas from CT and cryosection data. Phys Med Biol. 2007;52:577-87. https://doi:10.1088/0031-9155/52/3/003.

5. White DR, Griffith RV, Wilson IJ. ICRU Report 46: Photon, electron, proton, and neutron interaction data for body tissues. Journal of the International Commission on Radiation Units and Measurements. 1992;os24.

6. Maughan RL, Chuba PJ, Porter AT, Ben-Josef E, Lucas DR. The elemental composition of tumors: kerma data for neutrons. Med Phys. 1997;24:1241-4. https://doi:10.1118/1.598144.

7. Thomson RM, Tedgren AC, Williamson JF. On the biological basis for competing macroscopic dose descriptors for kilovoltage dosimetry: cellular dosimetry for brachytherapy and diagnostic radiology. Phys Med Biol. 2013;58:1123-50. https://doi:10.1088/0031-9155/58/4/1123.
